# Fungal-Derived tRNAs Are Expressed and Aminoacylated in Orchid Mitochondria

**DOI:** 10.1093/molbev/msaf025

**Published:** 2025-01-30

**Authors:** Jessica M Warren, Luis F Ceriotti, M Virginia Sanchez-Puerta, Daniel B Sloan

**Affiliations:** Biodesign Institute and School of Life Sciences, Arizona State University, Tempe, AZ, USA; Howard Hughes Medical Institute, Chevy Chase, MD, USA; IBAM, Universidad Nacional de Cuyo, CONICET, Facultad de Ciencias Agrarias, Mendoza, Argentina; Facultad de Ciencias Exactas y Naturales, Universidad Nacional de Cuyo, Mendoza, Argentina; IBAM, Universidad Nacional de Cuyo, CONICET, Facultad de Ciencias Agrarias, Mendoza, Argentina; Facultad de Ciencias Exactas y Naturales, Universidad Nacional de Cuyo, Mendoza, Argentina; Department of Biology, Colorado State University, Fort Collins, CO, USA

**Keywords:** horizontal gene transfer, tRNA, aminoacylation, mitochondrial genome

## Abstract

Plant mitochondrial genomes (mitogenomes) experience remarkable levels of horizontal gene transfer, including the recent discovery that orchids anciently acquired DNA from fungal mitogenomes. Thus far, however, there is no evidence that any of the genes from this interkingdom horizontal gene transfer are functional in orchid mitogenomes. Here, we applied a specialized sequencing approach to the orchid *Corallorhiza maculata* and found that some fungal-derived tRNA genes in the transferred region are transcribed, post-transcriptionally modified, and aminoacylated. In contrast, all the transferred protein-coding sequences appear to be pseudogenes. These findings show that fungal horizontal gene transfer has altered the composition of the orchid mitochondrial tRNA pool and suggest that these foreign tRNAs function in translation. The exceptional capacity of tRNAs for horizontal gene transfer and functional replacement is further illustrated by the diversity of tRNA genes in the *C. maculata* mitogenome, which also include genes of plastid and bacterial origin in addition to their native mitochondrial counterparts.

## Introduction

**Fig. 1.**
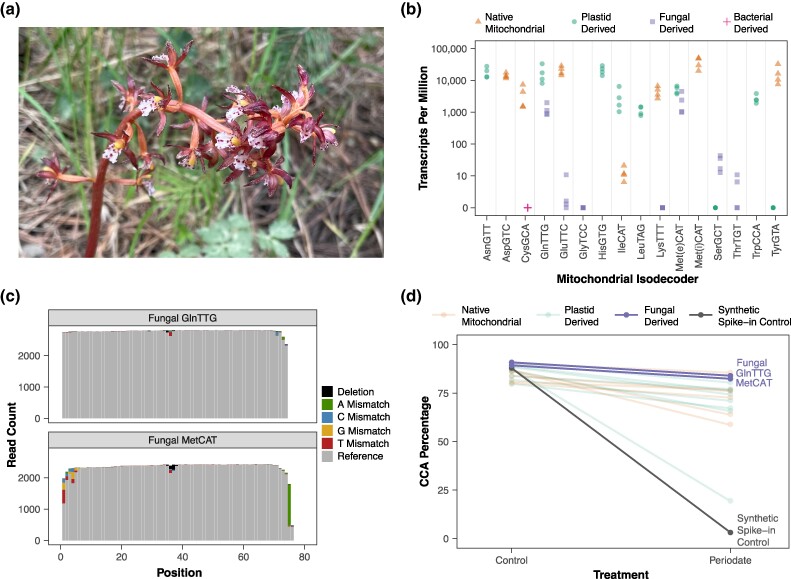
Expression, post-transcriptional modification, and aminoacylation of some fungal-derived tRNA genes in the *Corallorhiza maculata* mitogenome. a) Photograph of one of the *C. maculata* individuals used in this study. b) Expression of mitochondrial-encoded tRNAs. Read abundances for each of four *C. maculata* biological replicates (two flowers from each of two plants) are quantified as transcripts (reads) per million mapped to organellar reference sequences. Data are only plotted from control (no periodate) libraries and for reads that mapped uniquely to a reference sequence. The fungal-derived MetCAT was arbitrarily included in the Met(e)CAT (elongator) category, as the initiator/elongator distinction does not apply to fungal mitogenomes. Four plastid-derived tRNA genes in the *C. maculata* mitogenome (AsnGTT, CysGCA, PheGAA, and ThrTGT) were excluded from the reference because they are identical in sequence to their plastid-encoded counterparts and, therefore, indistinguishable in mapping. c) Read coverage and sequence variants in the two fungal-derived tRNA genes with substantial read abundance. Raw read counts are summed across the four control (no periodate) libraries after excluding reads that are truncated at the 3′ end (lacking >7 nt). d) Periodate treatment indicates high levels of aminoacylation for most expressed mitochondrial tRNAs, including the fungal-derived GlnTTG and MetCAT genes. Reported values are averaged across four replicates and represent the percentages of reads with intact CCA tails after excluding reads that lacked more than just a single 3′ nucleotide. Only genes with >30 reads per library are shown. The synthetic spike-in control shows that tRNAs not protected by an amino acid experience near complete removal of 3′ nucleotides. The retention of intact CCA tails following periodate treatment in most mt-tRNAs (including the two fungal-derived tRNAs with substantial expression levels) indicates high levels of aminoacylation. The one notable exception with little retention of the 3′ nucleotide is the plastid-derived tRNA-IleCAT that may be acting as a *nad7* t-element. The fungal-derived MetCAT percentages do not include reads that were only post-transcriptionally modified with a CA addition (instead of CCA). The CA-tailed reads exhibited much lower retention of the 3′ nucleotide after periodate treatment (supplementary table S2, Supplementary Material online).

Plant mitochondrial genomes (mitogenomes) are evolutionary hotspots for horizontal gene transfer (HGT), with high rates of organelle fusion and recombination resulting in transferred sequences from diverse sources, including plastids, the mitochondria of other plants, and even bacteria (Rice et al. 2013; Knie et al. 2015; Sanchez-Puerta et al. 2017). Although most transferred sequences are likely nonfunctional and readily lost, tRNA genes are exceptional in that they have frequently retained function in the recipient mitogenome (Joyce and Gray 1989; Marchfelder et al. 1990; Kitazaki et al. 2011), sometimes replacing the native mitochondrial copy (Marchfelder et al. 1990; Duchêne and Maréchal-Drouard 2001; Warren and Sloan 2020). Recently, another source of HGT was discovered with the report that mitochondrial DNA from a lineage of pathogenic fungi (Ustilaginomycetes) has been inserted into the mitogenome of orchids (Sinn and Barrett 2020; Valencia-D et al. 2023). A diverse clade of epidendroid orchids share a large (∼8 kb) insertion that was likely acquired 28 to 43 Mya (Valencia-D et al. 2023). In addition, the mitogenomes of two more distantly related orchid genera (*Apostasia* and *Vanilla*) contain a smaller DNA fragment (<300 bp) derived from the same fungal lineage. The history that has yielded these fragments in disparate parts of the Orchidaceae is unclear, and multiple evolutionary scenarios have been proposed (Sinn and Barrett 2020; Valencia-D et al. 2023). The large fungal-derived sequence in epidendroid orchids contains up to eight mitochondrial tRNA (mt-tRNA) genes, whereas the smaller fragment in *Apostasia* and *Vanilla* contains only three of these eight tRNA genes. Although expression of these genes was not detected (Sinn and Barrett 2020), tRNAs are notoriously difficult to sequence with conventional RNA-seq methods (Wilusz 2015; Padhiar et al. 2024). Therefore, it remains unclear whether this case of interkingdom HGT has functionally altered the make-up of the orchid mitochondrial tRNA pool. To address this question, we applied a specialized tRNA-seq strategy (Watkins et al. 2022) that enables robust detection of tRNAs to the coralroot orchid *Corallorhiza maculata* (Fig. 1a), a non-photosynthetic species that relies entirely on mycorrhizal fungi for carbon acquisition (mycoheterotrophy). We coupled this approach with a chemical pretreatment (sodium periodate followed by sodium tetraborate) that distinguishes between aminoacylated and uncharged tRNAs based on whether an amino acid is present to protect the tRNA from removal of its terminal 3′ nucleotide (Evans et al. 2017; Davidsen and Sullivan 2024). Determining the aminoacylation state for the foreign mt-tRNAs is essential for understanding whether they are recognized and charged by orchid aminoacyl-tRNA synthetases (aaRS) and, thus, have the potential to function in translation.

### The *C. maculata* Mitogenome Contains tRNA Genes of Diverse Origins

The assembled mitogenome of *C. maculata* is a posterchild for tRNA HGT, with 16 plastid-derived, one bacterial-derived, seven fungal-derived, and seven native mt-tRNA genes (supplementary table S1, Supplementary Material online). Other orchids have as many as eight fungal-derived tRNA genes, but the tRNA-Val gene is not found in *C. maculata*. The one bacterial gene (tRNA-Cys) has been identified in numerous angiosperms, including some in which it is expressed and aminoacylated (Kitazaki et al. 2011). However, we did not detect any expression of this gene in *C. maculata* (Fig. 1b). The plastid-derived mt-tRNA gene set represents 12 different anticodons and includes many genes that were anciently transferred during seed plant evolution (Richardson et al. 2013). It also includes expressed tRNAs that are not typically plastid-derived (or in some cases present at all) in plant mitogenomes, including tRNA-Gln, tRNA-Ile, and a copy of tRNA-Leu with partially disrupted base-pairing in its acceptor stem. We detected expression of all seven of the native mt-tRNAs. Although raw read abundances from tRNA-seq data should be interpreted cautiously due to large biases in the sequencing process (Warren et al. 2021a), it is noteworthy that the native tRNA-Ile was only detected at very low levels, whereas transcripts from the plastid-derived tRNA-Ile genes were abundant (Fig. 1b). This observation suggests that *C. maculata* may be undergoing a functional replacement of the native tRNA-Ile with its plastid-derived counterpart. The values reported in Fig. 1b for plastid-derived tRNA-Ile gene expression are summed across three gene copies (supplementary table S1, Supplementary Material online). Two of these copies are identical and account for 85% of the reads. The third differs by only a single substitution at the discriminator base position and may be functioning as a t-element (Forner et al. 2007) involved in 3′ processing of *nad7* rather than in translation (see Fig. 1d and supplementary fig. S2, Supplementary Material online [Scaffold 3]).

Despite the gain of so many mt-tRNA genes via HGT, *C. maculata* does not contain a minimally sufficient set of tRNA genes in its mitogenome to support translation and must presumably import nuclear-encoded tRNAs from the cytosol like other angiosperms (Schneider 2011). In contrast to the complex mix of mt-tRNA genes, the *C. maculata* plastome has the conventional angiosperm set of 30 tRNAs, all of which appear to be expressed at substantial levels (supplementary fig. S1, Supplementary Material online). Therefore, despite being an obligate heterotroph (i.e. non-photosynthetic), *C. maculata* shows no signs of the plastid tRNA gene loss that is often observed in plants with a more ancient history of heterotrophy (Wicke and Naumann 2018).

### Fungal-Derived tRNA Genes in the *C. maculata* Mitogenome Are Transcribed, Post-Transcriptionally Modified, and Aminoacylated

Our tRNA-seq analysis detected transcripts from five of the seven fungal-derived tRNA genes, although only two of them (tRNA-Gln and tRNA-Met) had substantial read abundances (Fig. 1b). For all five of these genes, we identified transcripts with a 3′ CCA tail, which is a typical feature of mature tRNAs. This tail is not genomically encoded in any of the genes and is presumably added post-transcriptionally by a tRNA nucleotidyltransferase (von Braun et al. 2007). Mature tRNAs also undergo extensive post-transcriptional base modifications (Suzuki 2021), some of which cause nucleotide misincorporations and deletions during reverse transcription (RT) (Clark et al. 2016; Behrens et al. 2021; Ceriotti et al. 2024). In the two fungal-derived tRNAs with suitable read depth for analysis, we found an increase in sequence variants around position 37 (Fig. 1c), which is immediately 3′ of the anticodon and a known site for base modifications in many tRNAs (Schweizer et al. 2017). However, the overall misincorporation rate for both of these tRNAs was low, including at sites such as 26G that show signatures in many (but not all) plant mt-tRNAs (Ceriotti et al. 2024), and the absence of large internal drops in coverage suggests that RT was not inhibited by “hard-stop” modifications (Fig. 1c). The sequence variants at the 5′ and 3′ ends of tRNA-Met (Fig. 1c) are likely mapping artifacts resulting from variation in transcript length. We found that many tRNA-Met reads are tailed with the post-transcriptional addition of CA instead of CCA. Because the preceding base (i.e. the discriminator base) is a C, both modifications result in a CCA tail but with different endpoints relative to the reference gene model. RT-mediated terminal transferase activity at the 5′ end of these tRNA-Met transcripts may also lead to artifactual mismatches relative to the reference sequence (Fig. 1c).

The fungal-derived tRNA-Gln and tRNA-Met transcripts retained intact CCA tails after periodate treatment at levels similar to other mitochondrial tRNAs (Fig. 1d), indicating that their 3′ ends are protected by an amino acid. For tRNA-Met, the retention of the 3′ nucleotide was much less common (2.6-fold lower on average) for transcripts that carried a post-transcriptional CA addition instead of CCA (see above), suggesting that the full-length tRNA-Met sequence is important for aminoacylation (supplementary table S2, Supplementary Material online). Inclusion of a synthetic spike-in control tRNA confirmed that the periodate treatment was effective in removing the 3′ nucleotide of uncharged tRNAs (Fig. 1d). Although precise estimates of aminoacylation levels for the other fungal-derived tRNAs are not feasible due to their extremely low read abundance, the small number of detected transcripts exhibited little or no retention of the 3′ nucleotide after periodate treatment, suggesting that they are generally not aminoacylated.

**Fig. 2.**
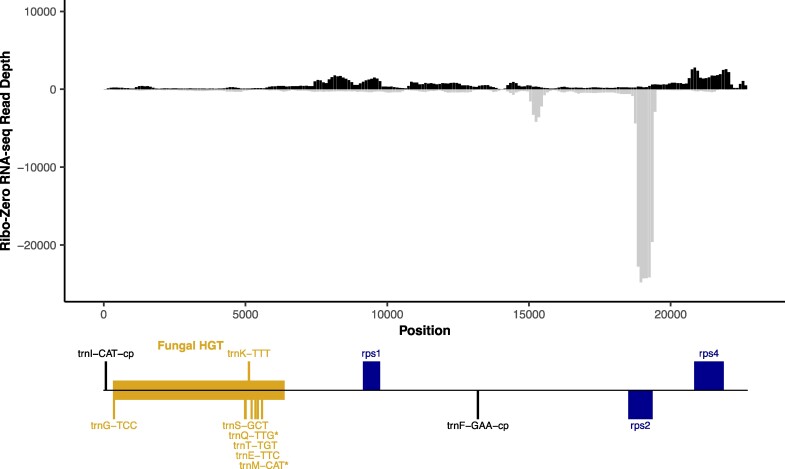
No evidence for functional expression of fungal-derived protein-coding genes in the *Corallorhiza maculata* mitochondrial genome. Transcript abundance was measured from rRNA-depleted (Ribo-Zero) RNA-seq mapped to the mitochondrial scaffold containing the fungal HGT region (Scaffold 8) from an assembly of total cellular DNA (see supplementary fig. S2, Supplementary Material online for mapping to other mitochondrial scaffolds). RNA and DNA were collected from the same individual (Plant 1). Depth is measured as average read sequence coverage (read count per position) for 100-bp windows across the length of the scaffold. Positive (black) and negative (gray) values indicate transcripts expressed from the forward and reverse orientations of the reference sequence, respectively. Mapping of sequencing reads from a second plant showed highly correlated read abundance across windows (*r* = 0.97 for log-transformed read depths). Annotated genes are indicated in the diagram below the *x* axis. Genes above and below the line correspond to being encoded on the forward and reverse strand of the reference sequence, respectively. The region that was horizontally acquired from fungi and associated tRNA genes are labeled as such and indicated in gold. In addition to the annotated tRNA genes in this region, it contains apparent pseudogenes derived from the fungal protein-coding genes *atp6*, *atp8*, *cox1*, *cox2*, *nad1*, and *nad4*. The tRNA genes outside this region on this scaffold are both plastid-derived. Because of column-based RNA purification, library size selection, and the challenges inherent in sequencing tRNAs, these RNA-seq libraries are not expected to capture mature tRNA expression. The two fungal-derived tRNA genes that were inferred from MSR-seq data to be expressed at substantial levels and aminoacylated are indicated with asterisks.

The fact that tRNA-Gln is expressed and aminoacylated in *C. maculata* is notable because this gene is found in both the larger and smaller fungal HGT fragments that are present within different lineages of the Orchidaceae (Sinn and Barrett 2020; Valencia-D et al. 2023). In contrast, the fungal-derived tRNA-Met gene is only found in the larger fragment, which also contains six protein-coding genes in *C. maculata*. None of these six genes retain an intact reading frame. The median mRNA-seq coverage across the fungal HGT region is similar to background levels for intergenic regions in the *C. maculata* mitogenome (64th percentile within the coverage distribution at intergenic sites), and no site in the fungal-derived fragment was in the top 5% of intergenic sites or reaches the expression level of any native protein-coding gene (Fig. 2 and supplementary fig. S2, Supplementary Material online). Therefore, it is unlikely that any of the transferred protein-coding genes retain function, and the expressed tRNAs may be the sole functional component of the fungal-derived HGTs.

Interestingly, neither of the two fungal-derived tRNAs that appear to be functionally expressed and aminoacylated expand the decoding capacity of the *C. maculata* mitogenome because native mitochondrial and/or plastid-derived tRNAs with the same anticodons are also present. Instead, the *C. maculata* mitogenome offers a striking example of tRNAs from diverse evolutionary origins being redundantly expressed in the same organelle (Fig. 1b). This redundancy may represent an intermediate state in a more general evolutionary process responsible for the exceptional propensity of plant mitogenomes for tRNA HGT and eventual functional replacement (Small et al. 1999; Warren and Sloan 2020; Warren et al. 2021b).

### *Corallorhiza maculata* Maintains Typical Plant Enzymatic Machinery for Aminoacylation of Organellar tRNA-Gln and tRNA-Met Despite tRNA HGT

The expression and apparent aminoacylation of the fungal-derived tRNA-Gln and tRNA-Met raise questions about the identity of the aaRSs charging these HGT tRNAs. Are they charged by a novel interaction with the native plant machinery or were some of the nuclear genes that encode mitochondrial-targeted enzymes also transferred from fungi to orchids? Plant nuclear genomes typically encode two separate sets of aaRSs for aminoacylating cytosolic versus organellar tRNAs (Duchêne et al. 2005). In *C. maculata*, we identified transcripts coding for typical cytosolic and organellar MetRS enzymes, and only the organellar MetRS was predicted to be targeted to the mitochondria (supplementary table S3, Supplementary Material online). In contrast, we did not find any MetRS sequences of fungal origin in the *C. maculata* transcriptome. Both findings suggest that a typical plant organellar MetRS is the sole enzyme for charging tRNA-Met in *C. maculata* mitochondria.

Aminoacylation of tRNA-Gln in plant and fungal mitochondria occurs via an indirect pathway in which the tRNA is first charged with Glu by a non-discriminating GluRS, and then Glu is converted to Gln by a tRNA-dependent amidotransferase (GatCAB in plant mitochondria and GatFAB in fungal mitochondria) (Pujol et al. 2008; Frechin et al. 2009; Araiso et al. 2014). All three subunits of the plant GatCAB complex were detected in the *C. maculata* transcriptome with predicted targeting to the organelles (supplementary table S3, Supplementary Material online), whereas no fungal-type GatFAB subunits were found. Additionally, we did not detect any fungal GlnRS/GluRS HGT or retargeting of the *C. maculata* cytosolic GlnRS enzyme to mitochondria (supplementary table S3, Supplementary Material online). These results suggest that the typical plant transamidation pathway is the only mechanism for charging tRNA-Gln in *C. maculata* mitochondria. However, we cannot confidently conclude that the HGT tRNAs are “correctly” loaded with the cognate amino acid, because the tRNA-seq method used in this study can only differentiate between charged and uncharged tRNAs and does not identify which amino acids are loaded onto the tRNAs. Understanding how these foreign tRNAs interact with plant enzymatic machinery despite more than a billion years of sequence divergence from their native tRNA counterparts is a fascinating area for future investigation. Species such as *C. maculata* and other orchids should provide excellent systems for characterizing the dynamics of these interkingdom HGTs and how the sequence and structure of interacting components evolve post-transfer.

## Methods

### Tissue Collection and RNA/DNA Extraction

Shoot tissue was collected from two *C. maculata* individuals growing <10 m apart near Fort Collins, CO, USA (40°33′49″N, 105°11′0″W). Samples were collected at 11:00 AM on June 4, 2024 and stored on ice until 1:30 PM when RNA extraction was performed using an acid-phenol method as previously described (Ceriotti et al. 2024). Two extractions were performed per plant, each using a whole flower sampled from near the shoot apex. An additional flower from each plant was used for DNA extraction (Qiagen DNeasy kit).

### Organelle Genome Sequencing and Analysis

Library construction and sequencing of *C. maculata* genomic DNA samples were performed by Novogene, using an NEBNext Ultra II DNA Library Prep Kit and a 2 × 150 bp run on an Illumina NovaSeq X platform. A subset of 15 M read pairs from the “Plant 1” sample was trimmed with Cutadapt v4.0 (Martin 2011) and assembled with SPAdes v4.0.0, using the meta option (Nurk et al. 2017). Putative mitogenome-derived scaffolds were extracted based on coverage depth (40 to 150×) and length (>1,000 bp) and then further screened for the presence of known plant mitochondrial gene sequences, resulting in a set of 23 scaffolds with a total length of 495,364 bp. To assemble a reference plastome, NOVOPlasty v4.3.1 (Dierckxsens et al. 2017) was seeded with the *accD* gene sequence from GenBank accession KM390016.1 (Barrett et al. 2014) and used to analyze the full set of trimmed reads from the Plant 1 sample. Organelle tRNA genes were identified with tRNAscan-SE 2.0 (Chan and Lowe 2019) followed by manual curation.

### Charged tRNA-seq

To measure tRNA expression and infer aminoacylation states based on retention of CCA tails, an MSR-seq protocol (Watkins et al. 2022) was performed on two biological replicates from each of two *C. maculata* plants both with and without periodate treatment. Library construction, sequencing, and data analysis (including quantification of read abundance, CCA-tailing, and base misincorporations) were performed as described previously (Ceriotti et al. 2024). The reference tRNA set for mapping included annotated tRNAs from *C. maculata* organellar genomes (see above), *Arabidopsis thaliana* nuclear tRNAs from the PlantRNA 2.0 database (Cognat et al. 2022), and a *Bacillus subtilis* tRNA-Ile that was synthesized and used as an internal “spike-in” control as described previously (Ceriotti et al. 2024).

### rRNA-Depleted RNA-seq

RNA samples (see above) from a single biological replicate for each *C. maculata* plant (samples 1A and 2A) were processed with a Qiagen RNeasy MinElute kit to remove contaminating genomic DNA and then used to generate strand-specific rRNA-depleted RNA-seq libraries (Illumina TruSeq Stranded Total RNA with Ribo-Zero Plant kit) and sequenced on a 2 × 150 bp run of an Illumina NovaSeq X platform. Library construction and sequencing were performed by Novogene. The resulting reads were trimmed with Cutadapt and mapped to *C. maculata* mitogenome scaffolds with Bowtie2 v2.2.5 using default parameters (Langmead and Salzberg 2012), and resulting alignments were analyzed with Samtools v1.17 (Li et al. 2009) and custom scripts to quantify mRNA and long noncoding RNA transcript abundance. Trimmed reads were also assembled de novo with Trinity v2.15.2 (Grabherr et al. 2011) and searched for homologs of aaRSs and GatCAB/GatFAB with NCBI TBLASTN v2.14.1+ (Camacho et al. 2009). Subcellular localization was predicted with TargetP v2.0 (Emanuelsson et al. 2000).

## Supplementary Material

msaf025_Supplementary_Data

## Data Availability

All raw sequencing reads are available via NCBI SRA (BioProject PRJNA1186809). Code and processed data files, including tRNA read counts, are available via GitHub (https://github.com/dbsloan/Corallorhiza_tRNAs). Transcriptome and genomic DNA assemblies are available via Zenodo (DOI: 10.5281/zenodo.14172377; https://zenodo.org/records/14172377).
