# Supplementary material for: Fungal-Derived tRNAs Are Expressed and Aminoacylated in Orchid Mitochondria
Source: Mol Biol Evol. 2025 Jan 30;42(2):msaf025. doi: 10.1093/molbev/msaf025 (PMC11827590; doi:10.1093/molbev/msaf025)
Supplement: msaf025_Supplementary_Data [file msaf025_supplementary_data.pdf]

Supplementary Material

Table S1. Summary of mitochondrial tRNA genes in *Corallorhiza maculata*

Amino Acid	Anticodon	Origin	Copies	Expression Detected	Notes	Sequence
Asp	GTC	Native	1	Yes		GGGGAAATAGCTCAGTTGGTTAGAGTGCTGGTCTGTCACGCCAGAAGTCGCGGGTTCGAACCCCGTTTTCCCCG
Cys	GCA	Native	1	Yes		GGCTAGGTAACATAATGGAAATGTATCGGACTGCAAATCCTGGAATGACGGTTCGACCCCGTCCTTGGCCT
Glu	TTC	Native	1	Yes		GTCCCTTTCGTCCAGTGGTTAGGACATCGTCTTTTCATGTCGAAGACACGGGTTCGATTCCCGTAAGGGATA
Ile	CAT	Native	1	Very Low		GGGCTTCTAGTTGAATTGGTTGAAACGTACCGCTCATAACGGGAATATTGTAGGTTCGAGCCCTACTAAGCCCA
Lys	TTT	Native	1	Yes		GGGTGTATAGCTCAGTTGGTAGAGCATTGGGCTTTTAACCTAATGGTCGCAGGTTCAAGTCCTGCTATACCCC
Met(i)	CAT	Native	1	Yes		AGCGGGGTAGAGGAATTGGTCGACTCATCAGGCTCATGACCTGAAGACTGCAGGTTCGAATCCTGTCCCCGCCT
Tyr	GTA	Native	1	Yes		GGGAGAGTGGCCGAGTGGTCAAAAGCGACAGACTGTAAATCTGTTGAAGGTTTTCTACGTAGGTTCGAATCCTGCCTCTCCCA
Asn	GTT	Plastid	1	Yes		TCCTCAATAGCTCAGCGGTAGAGCGGTCAGCTGTTAACTGACTGGTCGTAGGTTCAAATCCTACTTGGGGAG
Asn	GTT	Plastid	1	Not tested	Identical to copy in plastome	TCCTCAGTAGCTCAGTGGTAGAGCGGTTGGCTGTTAACTGACTGGTCGTAGGTTCGAATCCTACTTGGGGAG
Cys	GCA	Plastid	1	Not tested	Identical to copy in plastome	GGCGGCATGGCCAAGTGGTAAGGCGGGGGACTGCAAATCCTTTATCCCCAGTTCGAATCTGGGTGTCGCCT
Gln	TTG	Plastid	1	Yes		TGGGGCGTGGCCAAGCGGTAAGGCAACGGGTTTTGGTCCCGTTACTCGGAGGTTCGAATCCTTCCGTCCCAG
His	GTG	Plastid	1	Yes		GCGAATGTAGCCAAGTGGATCAAGGCAGTGGATTGTGAATCCACCATGCGCGGGTTCAATTCCCGTCGTTCGCC
Ile	CAT	Plastid	2	Yes		GCATCCATGGCTGAATGGTGAAAGCGCCCAACTCATAATTGGCCAATTTGTAGGTTCAATTCCTGCTGGATGCA
Ile	CAT	Plastid	1	Yes	Low aminoacylation rate; *nad7* t-element	GCATCCATGGCTGAATGGTGAAAGCGCCCAACTCATAATTGGCCAATTTGTAGGTTCAATTCCTGCTGGATGCG
Leu	TAG	Plastid	1	Yes	Divergent acceptor stem base-pairing	GCCGCCATGGTGAAATTGGTAGACACGCTGCTCTTAGGGAGCAGTGCTAGAGCATCTCGGTTCGAGTCCGAGTGGGGCAT
Met(e)	CAT	Plastid	1	Yes		ACCTACTTGACTCAGCGGTTAGAGTATCGCTTTCATACGGCGAGAGTCATTGGTTCAAATCCAATAGTAGGTA
Phe	GAA	Plastid	2	Not tested	Identical to copy in plastome	GTCGGGATAGCTCAGTTGGTAGAGCAGAGGACTGAAAATCCTCGTGTCACCAGTTCAAATCTGGTTCCTGACA
Ser	GCT	Plastid	1	No		GGAAAGATGGCTGAGTGGACTAAAGCGTCGGATTGCTAATCCGTTGTACGAATTATTCGTACCGAGGGTTTGAATCCCTCTCTCTCCG
Thr	TGT	Plastid	1	Not tested	Identical to copy in plastome	GCCCGCTTAGCTCAGAGGTTAGAGCATCGCATTTGTAATGCGATGGTCATCGGTTCGATTCCGATAGCCGGCT
Trp	CCA	Plastid	1	Yes		GCGCTCTTAGTTCAGTTCGGTAGAACGCGGGTCTCCAAAACCCGATGTCGTAGGTTCAAATCCTACAGAGCGTG
Tyr	GTA	Plastid	1	No		TGGCCGAGTGGTTCAAGGCGTAGCATTGTAACTGCTATGTAGACTTTTGTTTACCGAGGGTTCGAATCCTAGCTTGAG

Cys	GCA	Bacterial	1	No	GGAACCCTATCCAAGTGGCTAAGGCAGAAGTCTGCAAAACTTCTATTCTAAGGTTCGAATCCGACCGGTTCCT
Gln	TTG	Fungal	1	Yes	TAGGATGTCGTCTAAAGGAAAGACATCATTTTTTGGTAATGAGAATGGTTGTTCGATTCAACCCATCCTAG
Glu	TTC	Fungal	1	Very Low	GGTCTTTTAGTTTCATGGGAAAAACCTCGGTCTTCATCCGAGTGAGTAGGCGTTCGATTCGCCTAAAGATCA
Gly	TCC	Fungal	1	No	GCTAGTCTATTTTCATGGTAGAATCAGGTACTTCCAATACCCGGGCTTCGGTTCGATTCCTAATACTAGTA
Lys	TTT	Fungal	1	No	GAAGGTATAACTCAGTCGGTTAGAGTGCAATACTTTTCATATCGAGGCCGTAGGTTAGAATCCTACTACCTTTC
Met	CAT	Fungal	1	Yes	AGTACTTTAGTGTAAAGGTAGCACAGCAGATTCATGTCCTGCTAGACCAGTGTTCAAATCCTGGAATGTACTC
Ser	GCT	Fungal	1	Very Low	GGACAAACCGCTATTGGTGAAGTGTAGGCGATTGCTAATTGTCTGGAAAATTCAACCATTCCTGAGGAGTTCGAATCTCCTTTTGTCCG
Thr	TGT	Fungal	1	Very Low	GTCGACTTAGTTTCATGGTAGAATAGGTAACTTGTAATTACTTGGCACTAGTTCGATTCTAGTAGTCGGCA

Table S2. Differential retention of 3′ terminal nucleotide following periodate treatment in fungal-derived tRNA-Met transcripts with CCA vs CA tails.

	Proportion of Reads (Control Libraries)		Proportion of Reads (Periodate Libraries)		Periodate/Control Ratio	
**Replicate**	**CCA**	**CA**	**CCA**	**CA**	**CCA**	**CA**
1A	0.106	0.488	0.096	0.166	0.909	0.340
1B	0.117	0.422	0.092	0.141	0.788	0.334
2A	0.122	0.412	0.134	0.128	1.100	0.311
2B	0.055	0.158	0.044	0.063	0.809	0.400
**Mean**	**0.100**	**0.370**	**0.092**	**0.125**	**0.901**	**0.346**

Table S3. Targeting predictions for *Corallorhiza maculata* enzymes involved in charging of tRNA-Gln and tRNA-Met. Transcript and protein sequences are available via GitHub (https://github.com/dbsloan/Corallorhiza_tRNAs).

			TargetP Analysis		
**Gene**	**Arabidopsis Ref**	**Trinity Contig**	**Prediction**	**Mito Prob**	**Plastid Prob**
GatA	AT3G25660	TRINITY_DN17257_c0_g1_i12	Plastid	0.009	0.666
GatB	AT1G48520	TRINITY_DN6546_c1_g1_i3	Mito	0.913	0.019
GatC	AT4G32915	TRINITY_DN31742_c0_g1_i1	Mito	0.771	0.080
GlnRS (cytosolic)	AT1G25350	TRINITY_DN21878_c0_g5_i5	None	0.000	0.000
GluRS (organellar)	AT5G64050	TRINITY_DN17518_c0_g1_i5	Plastid	0.236	0.643
GluRS (cytosolic)	AT5G26710	TRINITY_DN951_c0_g1_i5	None	0.006	0.091
MetRS (cytosolic)	AT4G13780	TRINITY_DN72148_c1_g1_i1	None	0.000	0.000
MetRS (organellar)	AT3G55400	TRINITY_DN1188_c0_g1_i5	Plastid	0.323	0.595

Figure S1. Expression of plastid-encoded tRNAs. Read abundances for each of four *Corallorhiza maculata* biological replicates (two flowers from each of two plants) are quantified as transcripts (reads) per million mapped to organellar reference sequences. Data are only plotted from control (no periodate) libraries and for reads that mapped uniquely to the reference. Note that read abundance for AsnGTT, CysGCA, PheGAA, and ThrTGT could also reflect expression of mitochondrial copies because there are identical copies of these plastid genes inserted in the mitogenome.

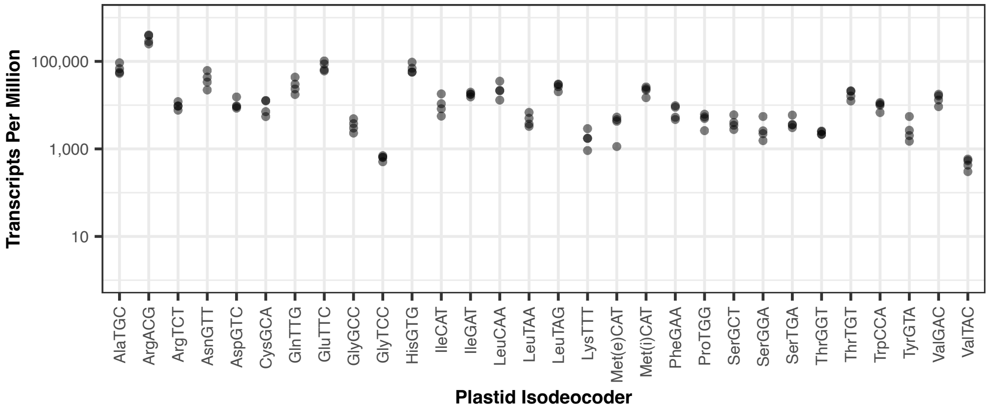

Figure S2. Transcript abundance measured from rRNA-depleted (Ribo-Zero) RNA-seq reads mapped to identified mitochondrial scaffolds from an assembly of total cellular DNA from *Corallorhiza maculata*. RNA and DNA were collected from the same individual (Plant 1). Depth is measured as average read sequence coverage (read count per position) for 100-bp windows across the length of the scaffolds. Positive and negative values indicate transcripts expressed in forward and reverse orientations relative to the reference sequence, respectively. Annotated genes are indicated in the diagram below the x-axis. Genes above and below the line correspond to being encoded on the forward and reverse strand of the reference sequence, respectively. The origins of tRNA gene are indicated as “native” (mitochondrial), “cp” (plastid intracellular gene transfer), and “bact” (bacterial horizontal gene transfer). Because of column-based RNA purification, library size selection, and the challenges inherent in sequencing tRNAs, these RNA-seq libraries are not expected to capture mature tRNA expression.

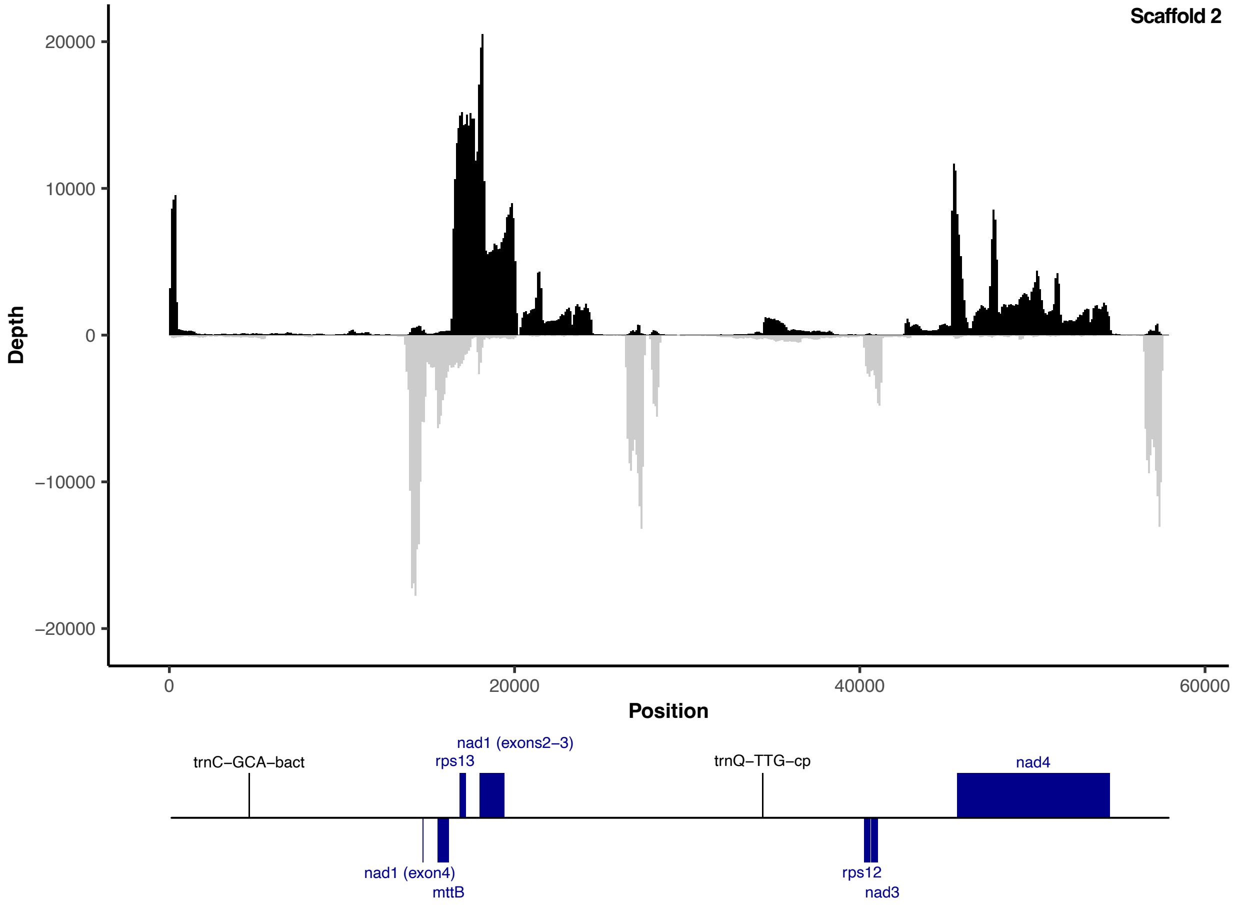

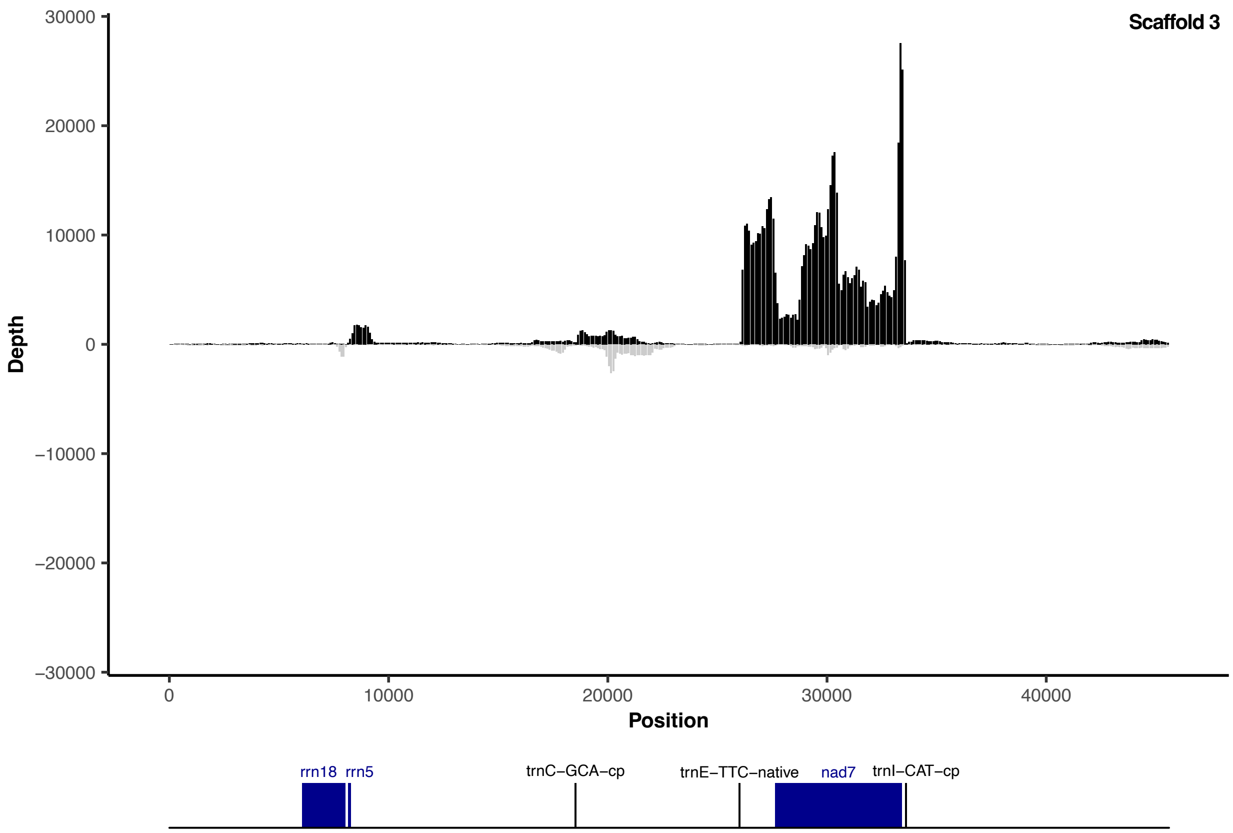

Scaffold 3
Depth
30000
20000
10000
0
−10000
−20000
−30000
0
10000
20000
30000
40000
Position
rrn18
rrn5
trnC−GCA−cp
trnE−TTC−native
nad7
trnI−CAT−cp

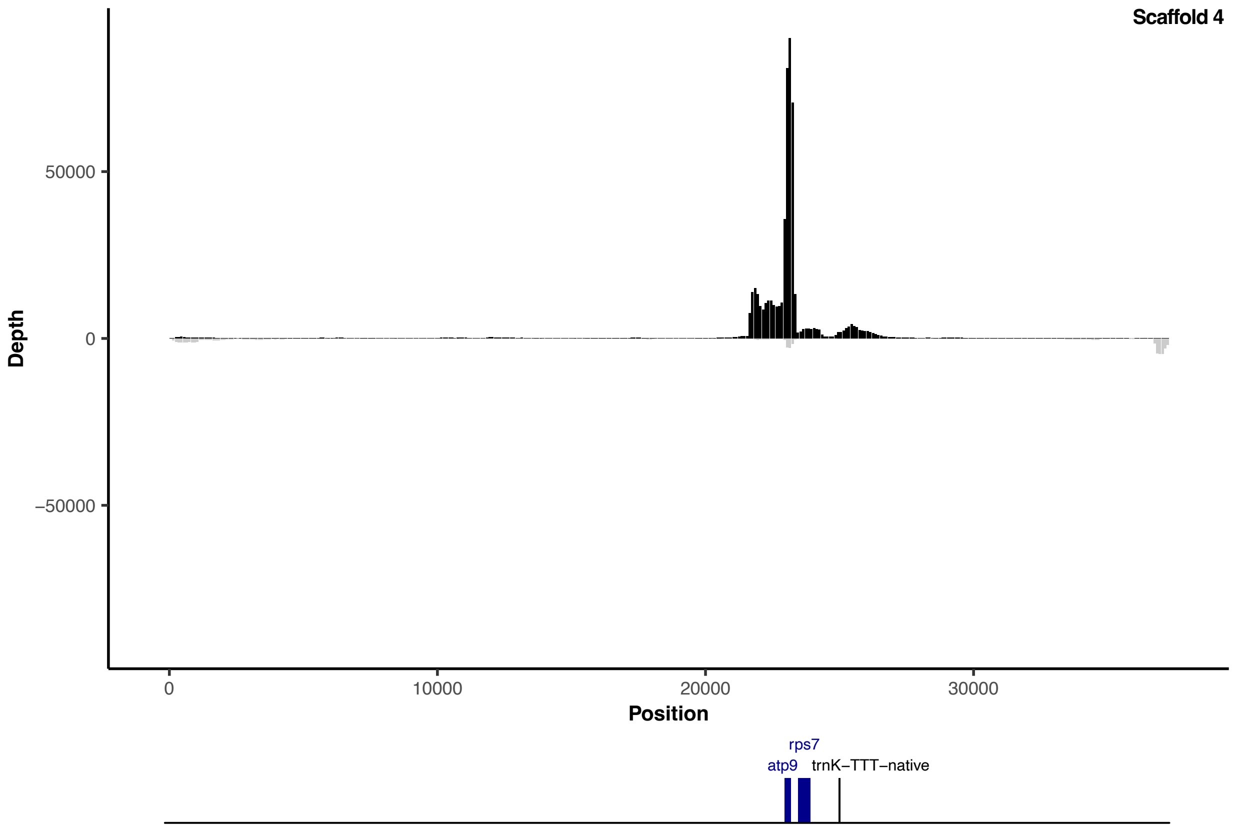

Scaffold 4
Depth
50000
0
−50000
0
10000
20000
30000
Position
rps7
atp9
trnK−TTT−native

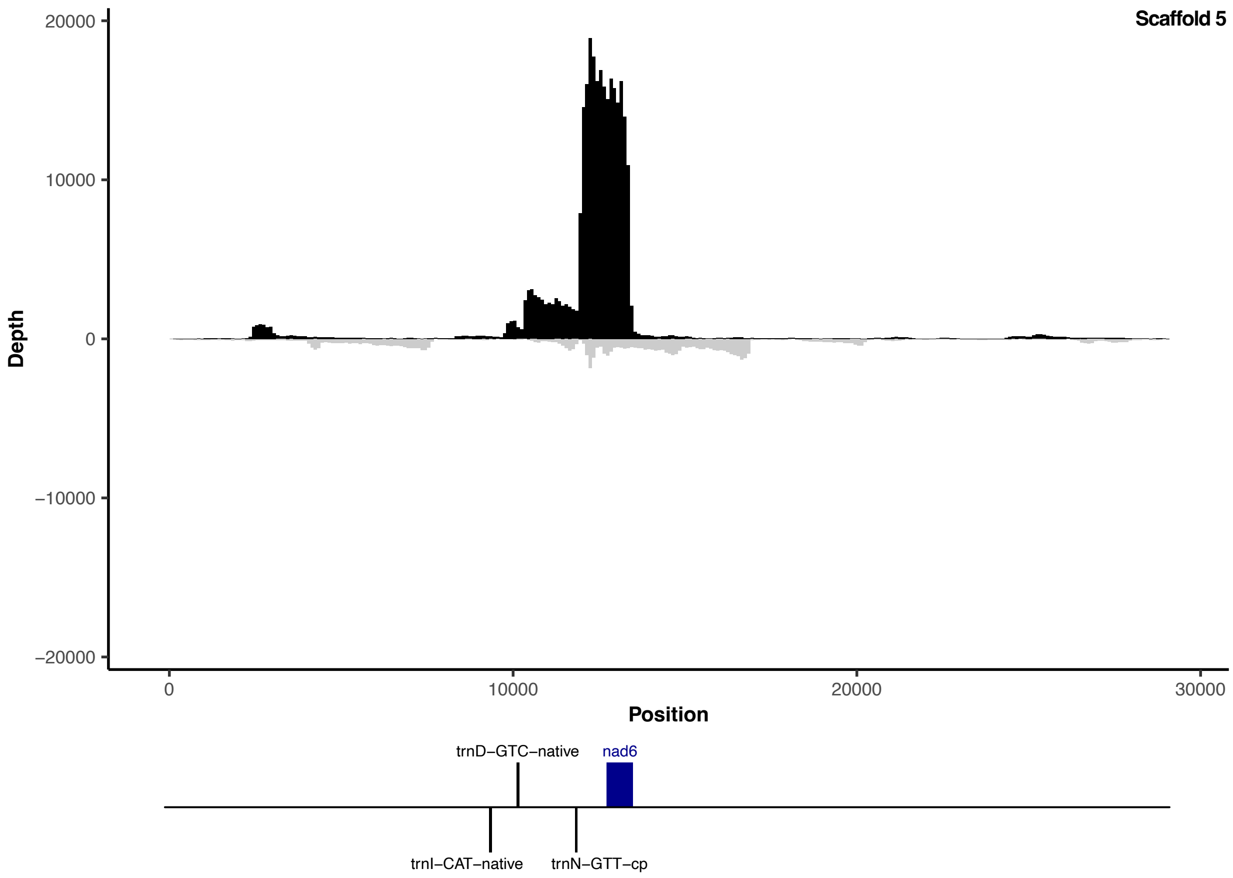

Scaffold 5
20000
10000
0
−10000
−20000
Depth
0
10000
20000
30000
Position
trnD−GTC−native
nad6
trnI−CAT−native
trnN−GTT−cp

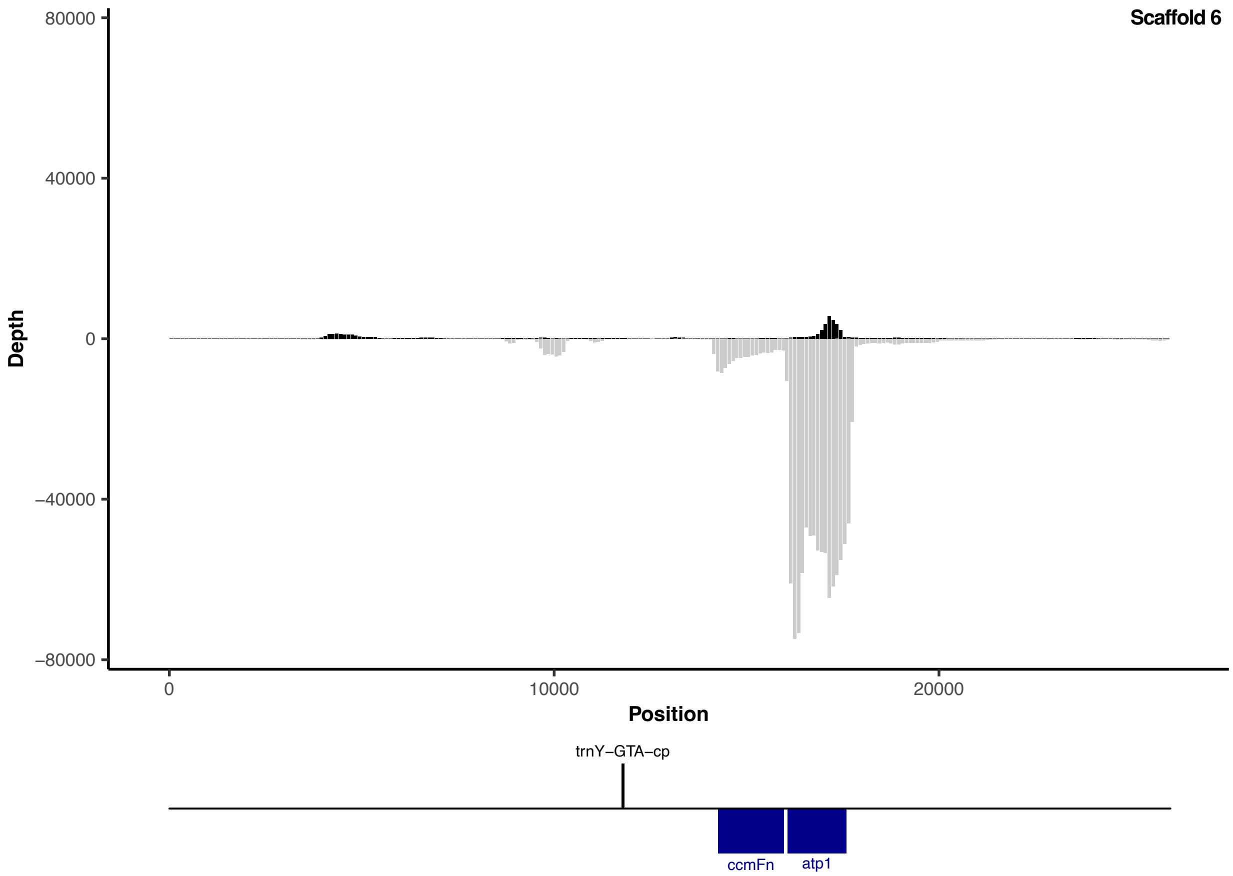

Scaffold 6
80000
40000
0
−40000
−80000
Depth
0
10000
20000
Position
trnY−GTA−cp
ccmFn
atp1

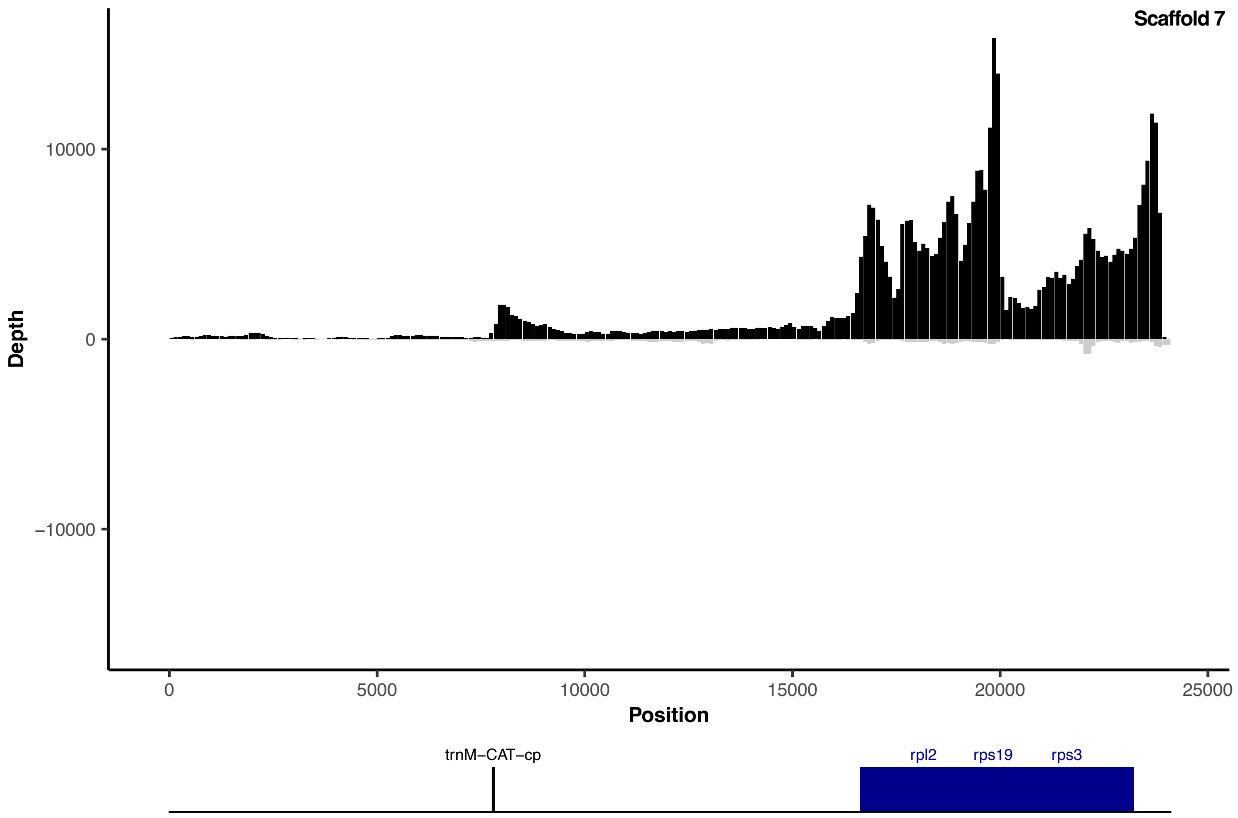
Scaffold 7
Depth
10000
0
−10000
0
5000
10000
15000
20000
25000
Position
trnM−CAT−cp
rpl2
rps19
rps3

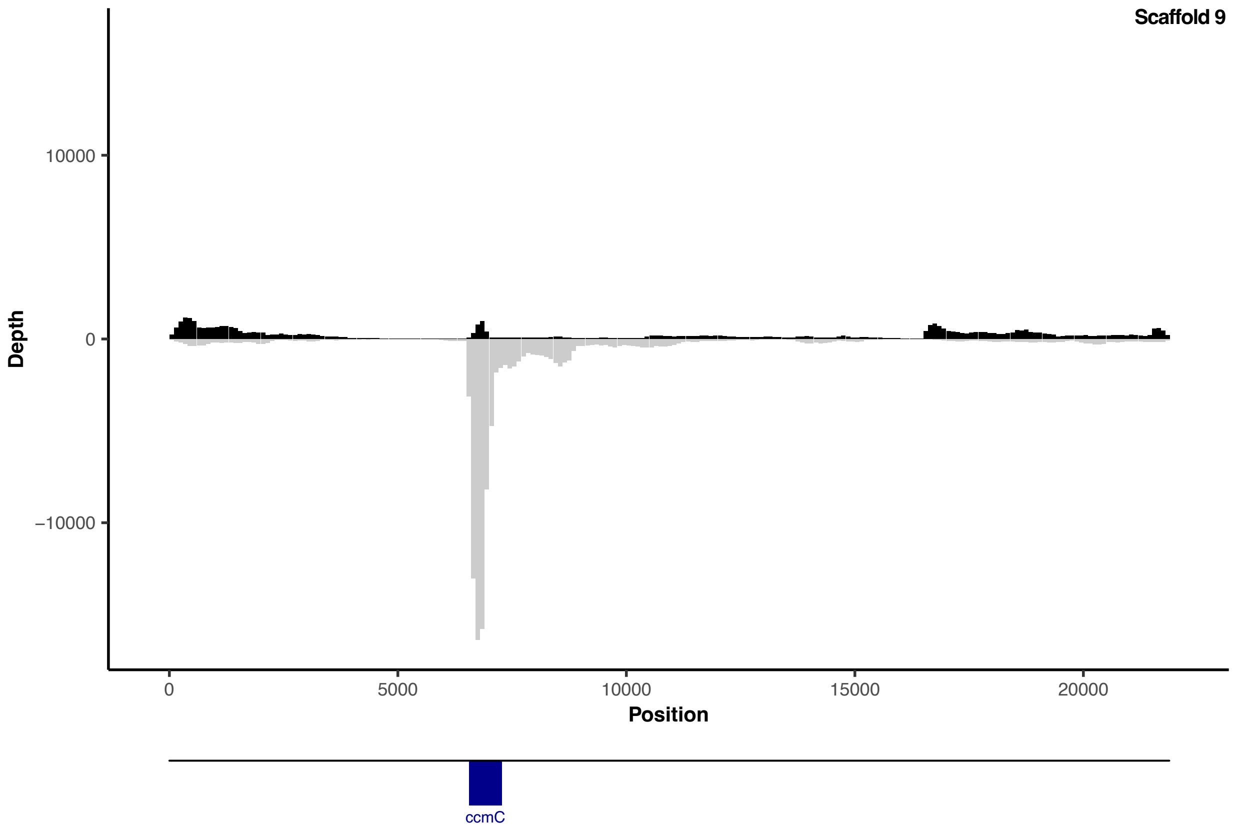

Scaffold 9
Depth
10000
0
−10000
0
5000
10000
15000
20000
Position
ccmC

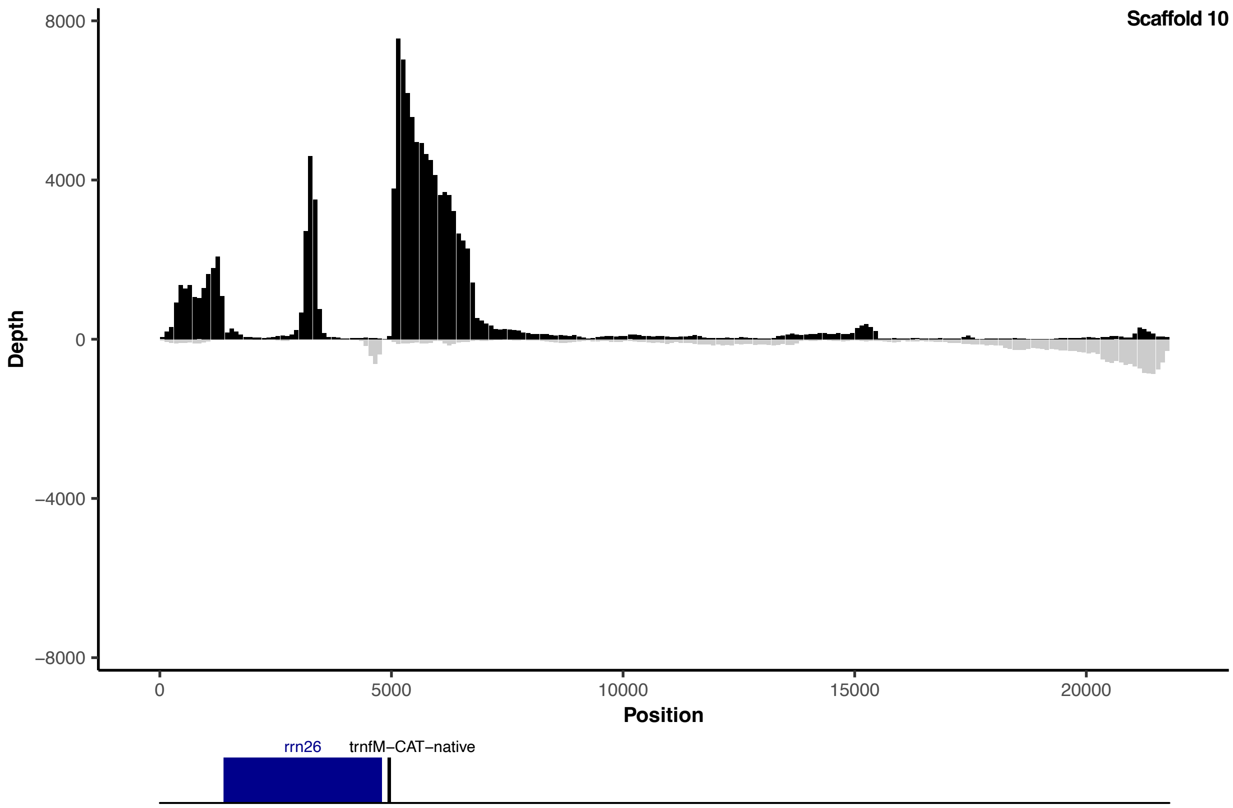

Scaffold 10
Depth
8000
4000
0
−4000
−8000
0
5000
10000
15000
20000
Position
rrn26
trnfM−CAT−native

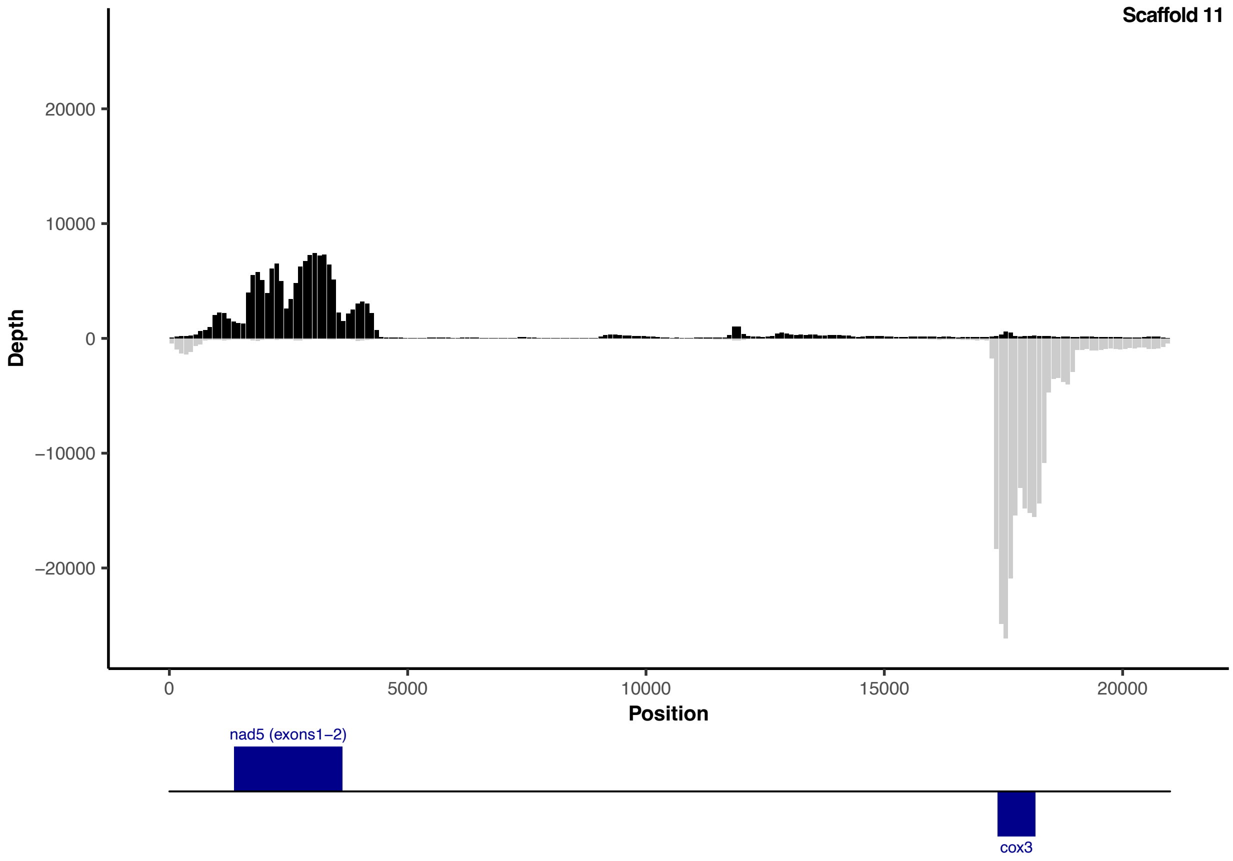
Scaffold 11
20000
10000
0
–10000
–20000
Depth
0
5000
10000
15000
20000
Position
nad5 (exons1–2)
cox3

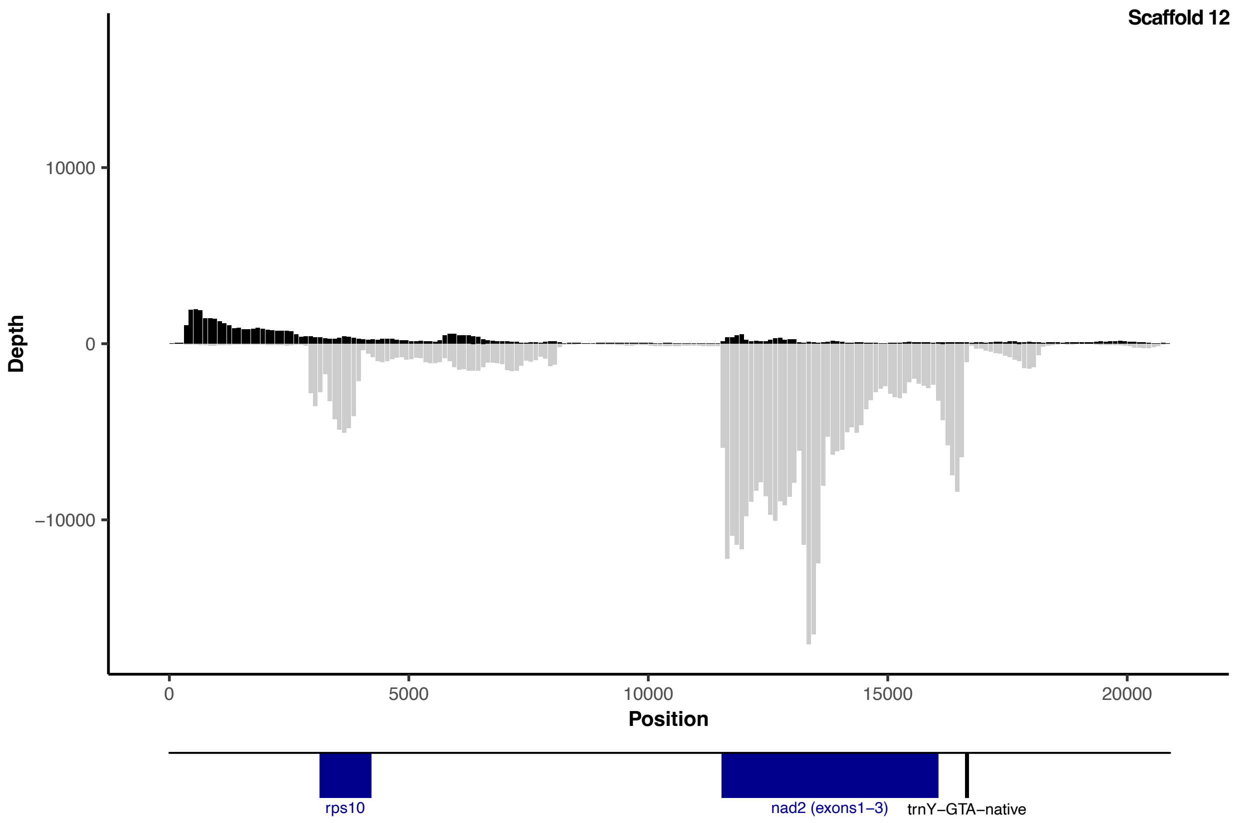
Scaffold 12
Depth
10000
0
−10000
0
5000
10000
15000
20000
Position
rps10
nad2 (exons1−3)
trnY−GTA−native

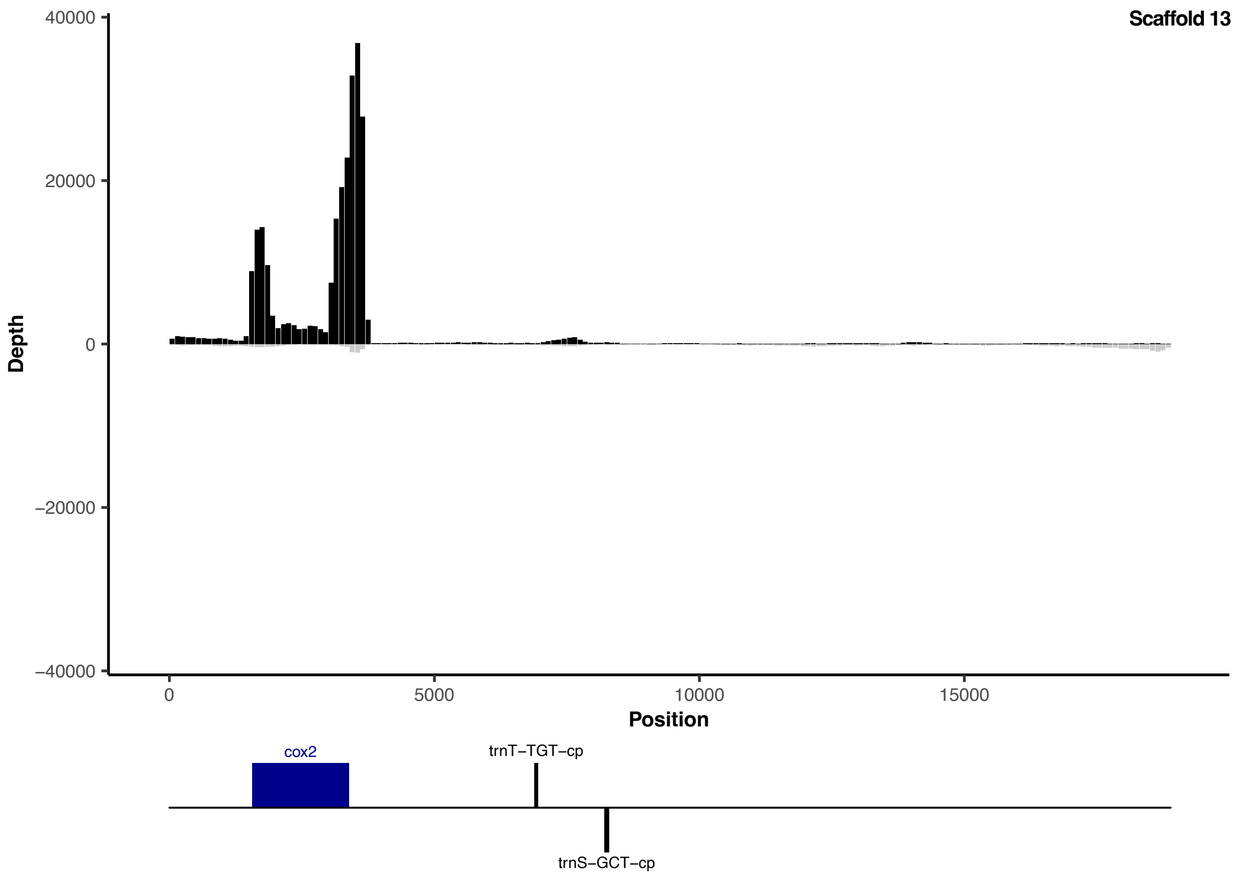

Scaffold 13
Depth
40000
20000
0
−20000
−40000
0
5000
10000
15000
Position
cox2
trnT−TGT−cp
trnS−GCT−cp

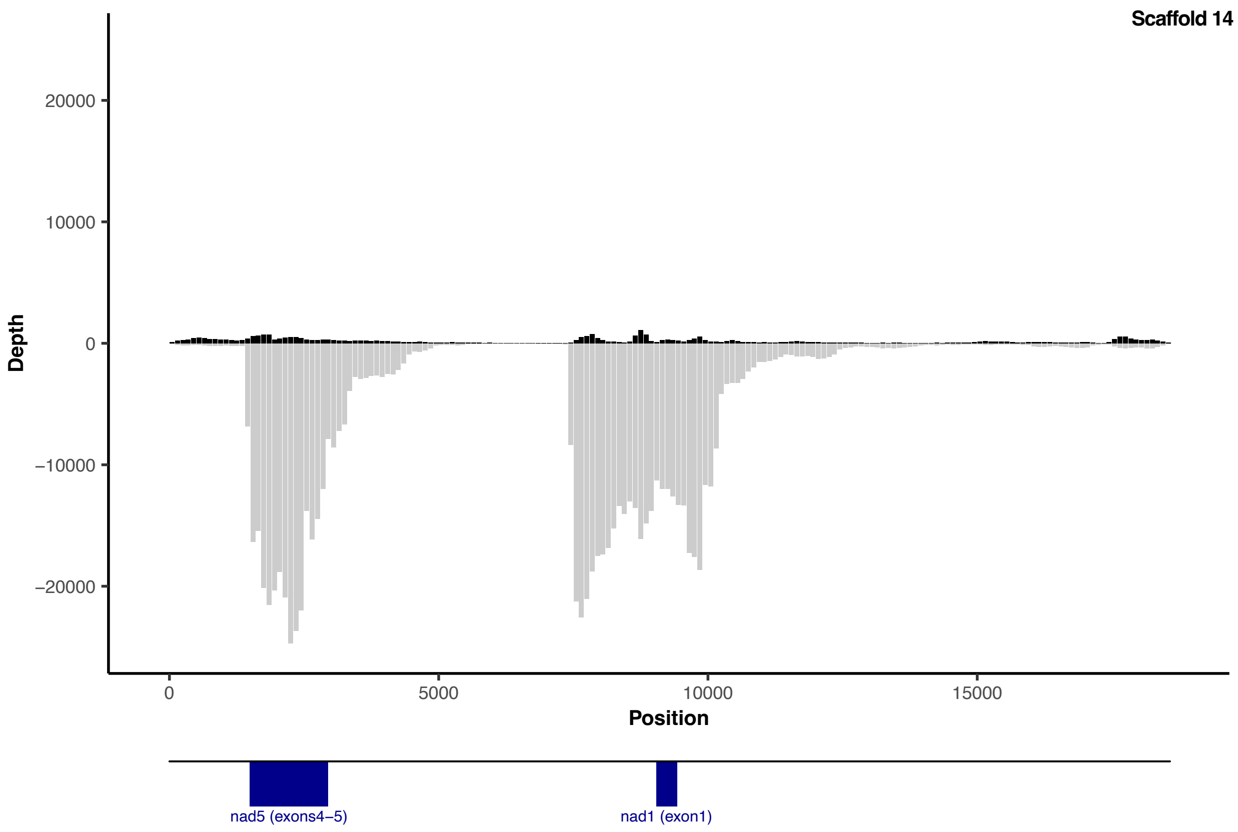

Scaffold 14
Depth
20000
10000
0
−10000
−20000
0
5000
10000
15000
Position
nad5 (exons4−5)
nad1 (exon1)

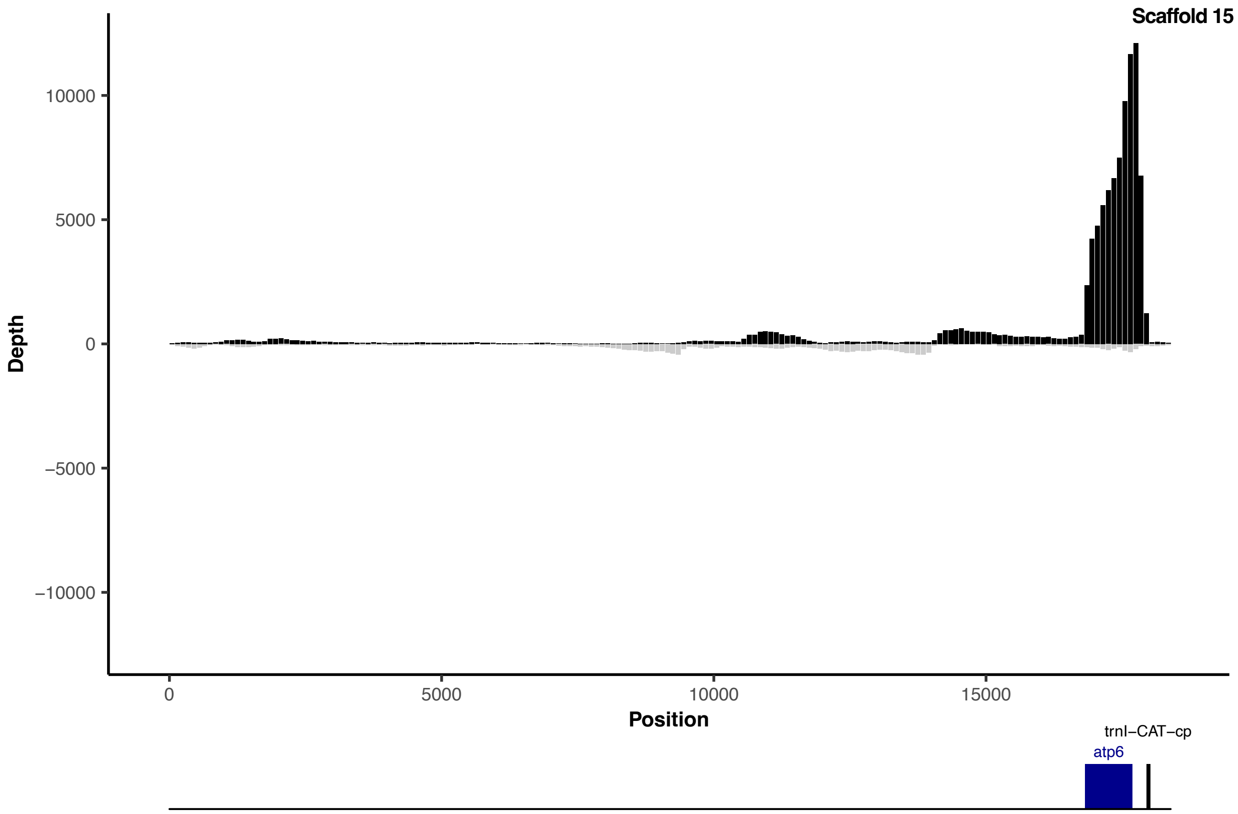
Scaffold 15
10000
5000
0
−5000
−10000
Depth
0
5000
10000
15000
Position
trnI−CAT−cp
atp6

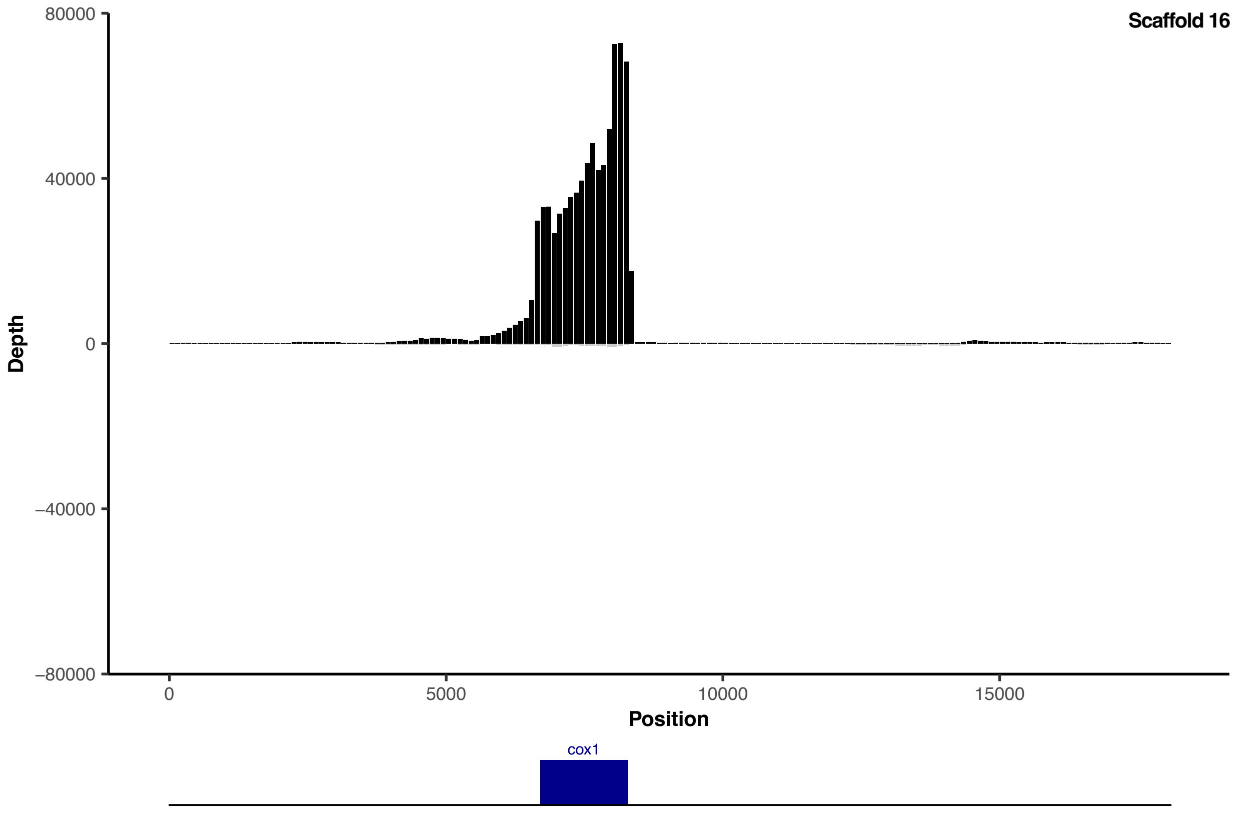

Scaffold 16
Depth
80000
40000
0
−40000
−80000
0
5000
10000
15000
Position
cox1

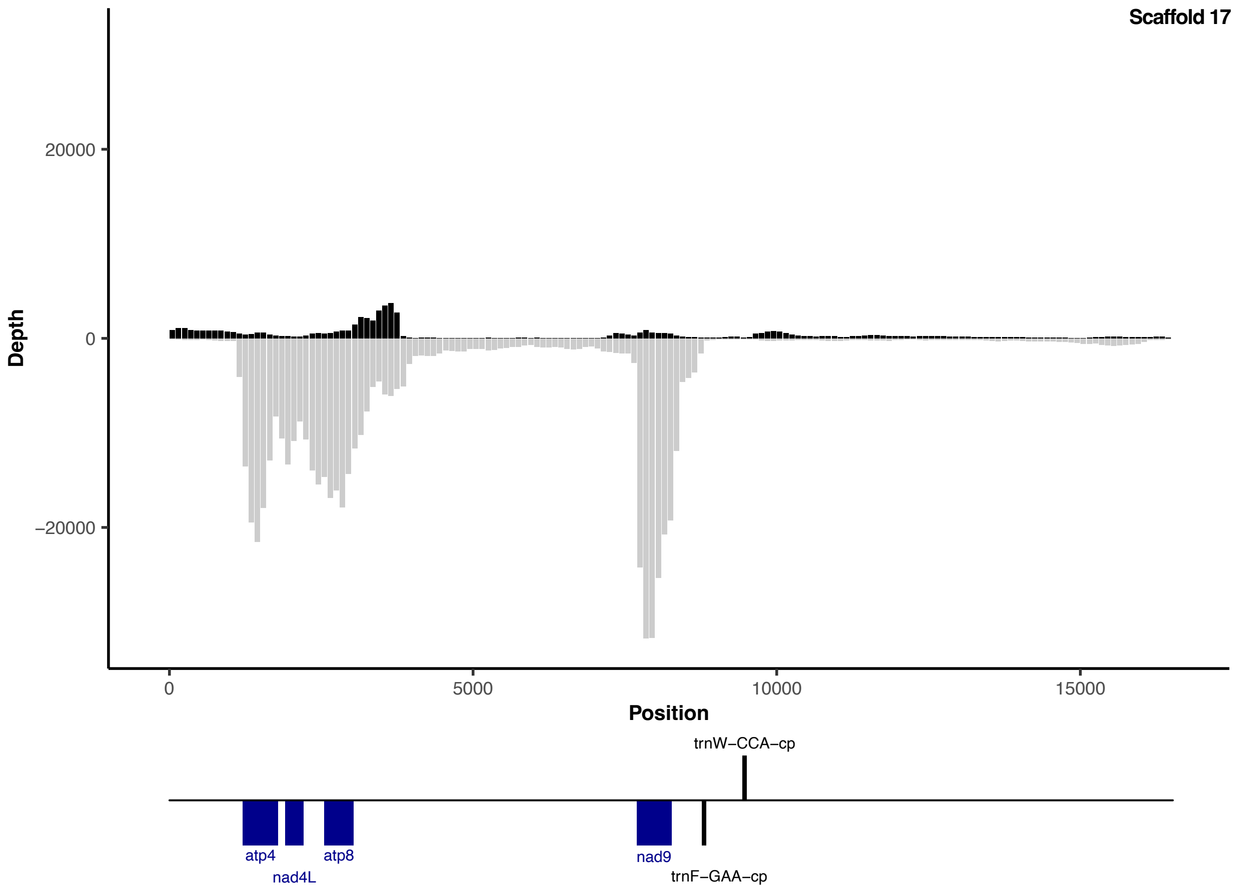
Scaffold 17
Depth
20000
0
−20000
0
5000
10000
15000
Position
trnW−CCA−cp
atp4
nad4L
atp8
nad9
trnF−GAA−cp

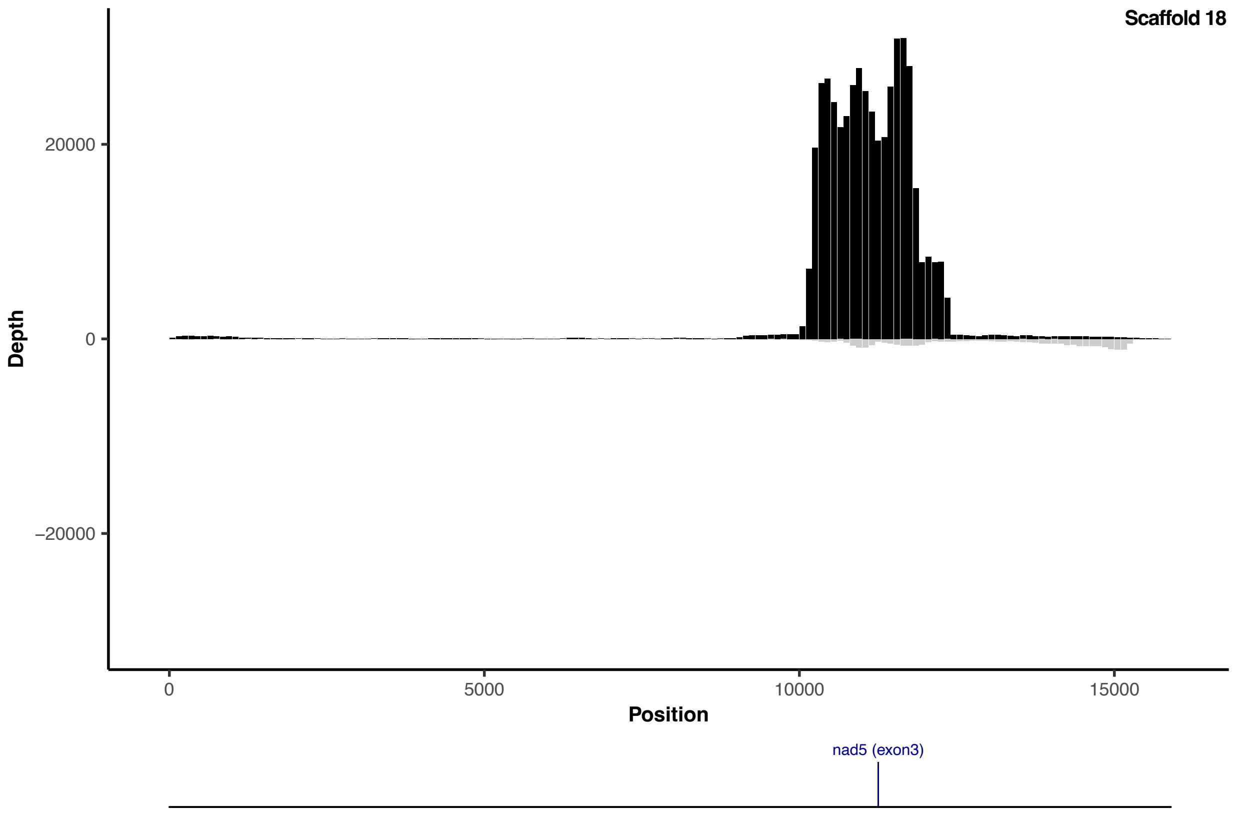
Scaffold 18
20000
0
−20000
Depth
0
5000
10000
15000
Position
nad5 (exon3)

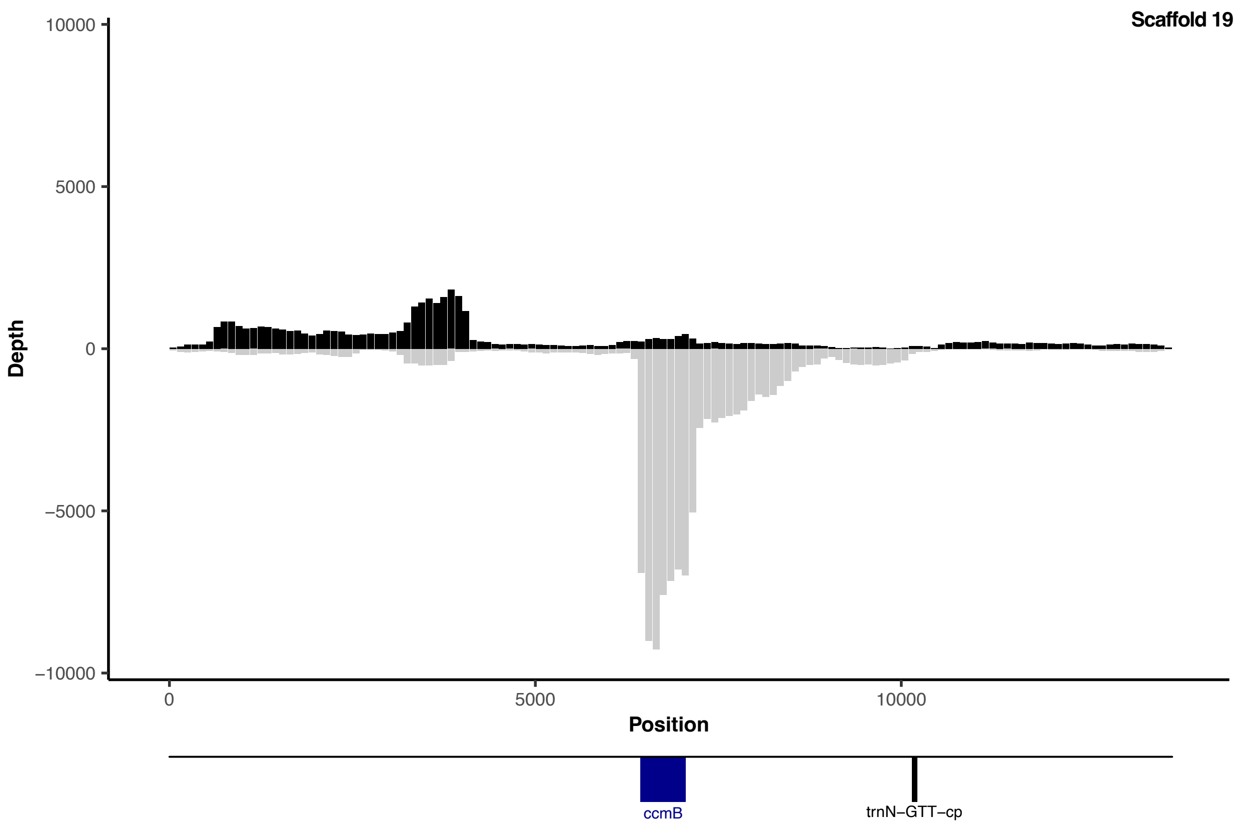

Scaffold 19
10000
5000
0
−5000
−10000
Depth
0
5000
10000
Position
ccmB
trnN−GTT−cp

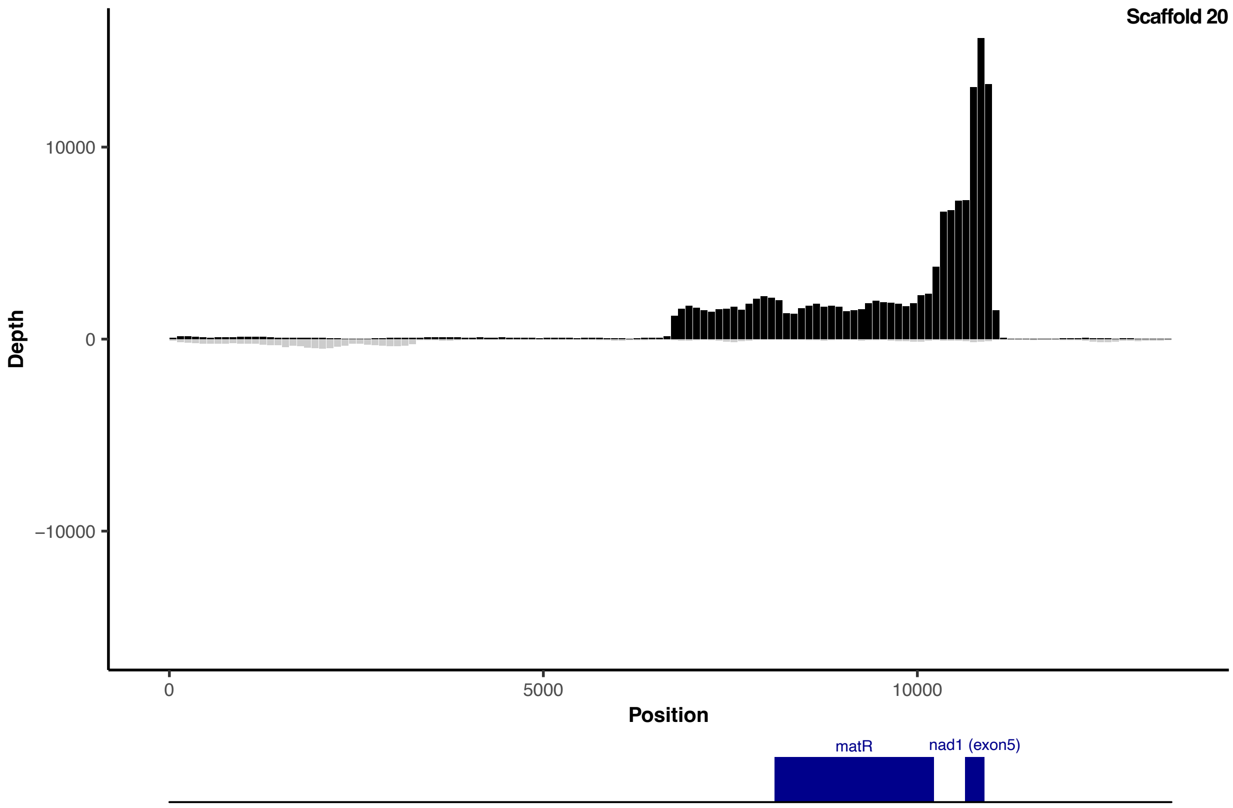

Scaffold 20
Depth
10000
0
−10000
0
5000
10000
Position
matR
nad1 (exon5)

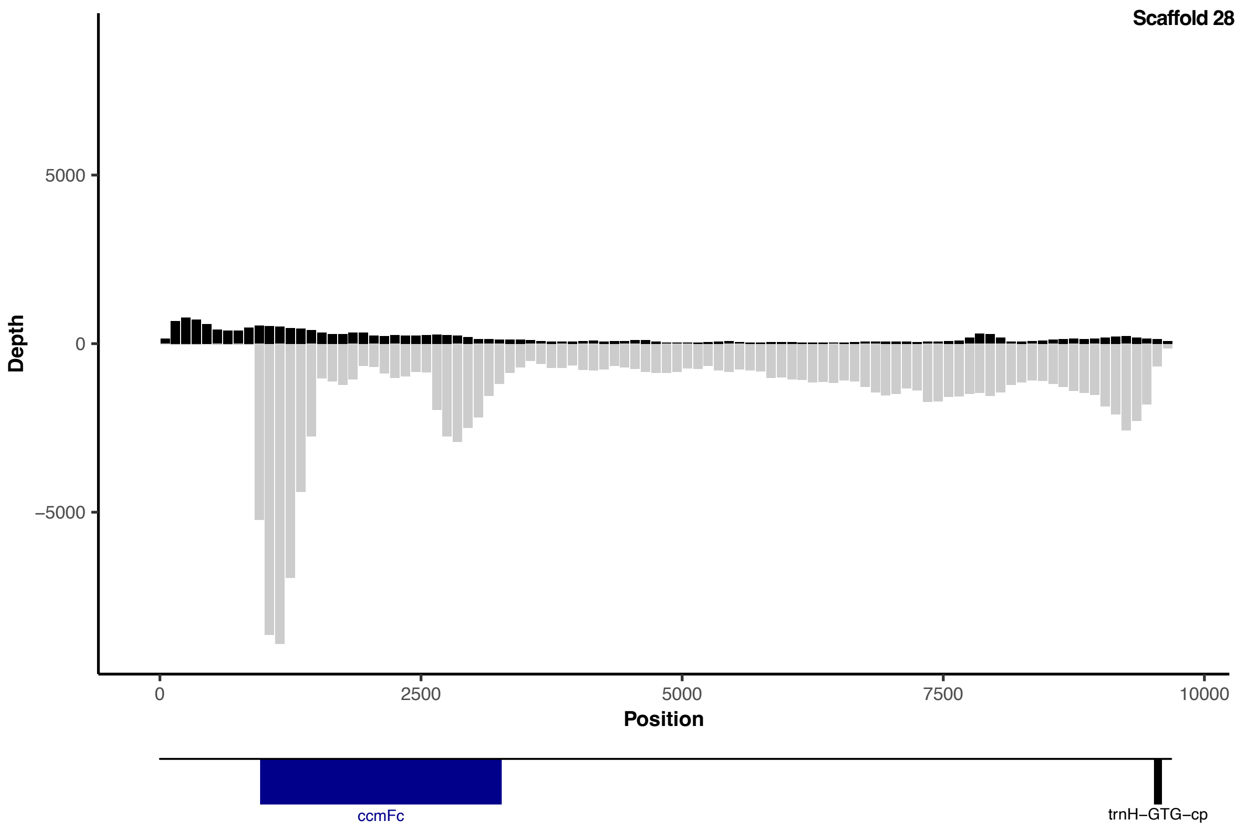
Scaffold 28
Depth
5000
0
−5000
0
2500
5000
7500
10000
Position
ccmFc
trnH−GTG−cp

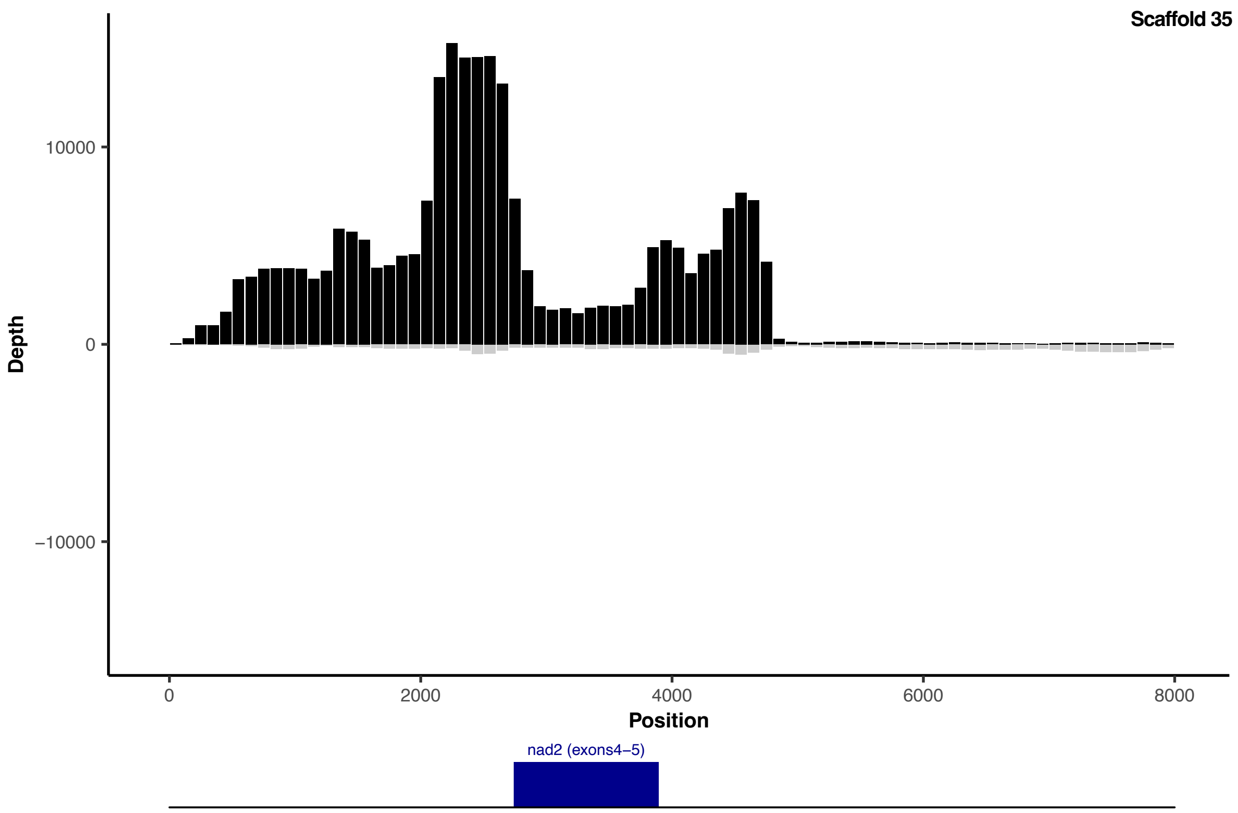
Scaffold 35
Depth
10000
0
−10000
0
2000
4000
6000
8000
Position
nad2 (exons4−5)

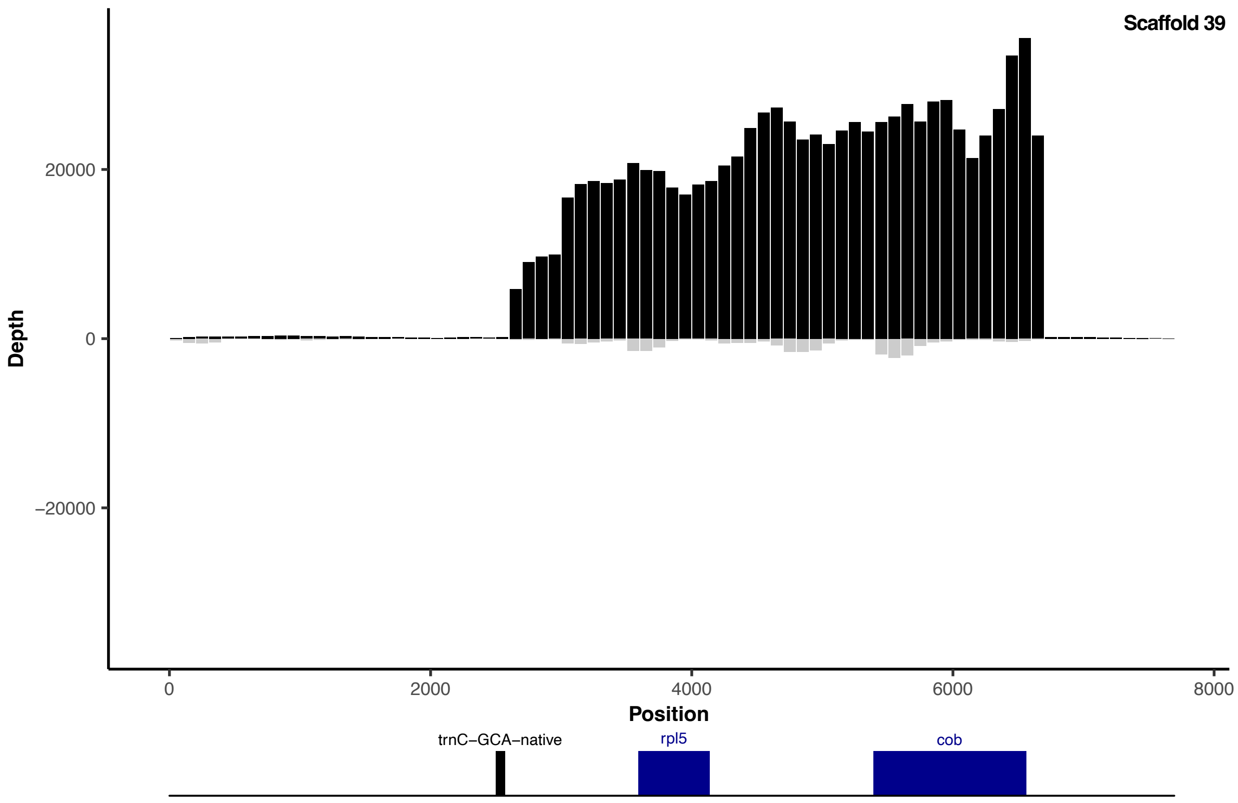

Scaffold 39
Depth
20000
0
−20000
0
2000
4000
6000
8000
Position
trnC−GCA−native
rpl5
cob

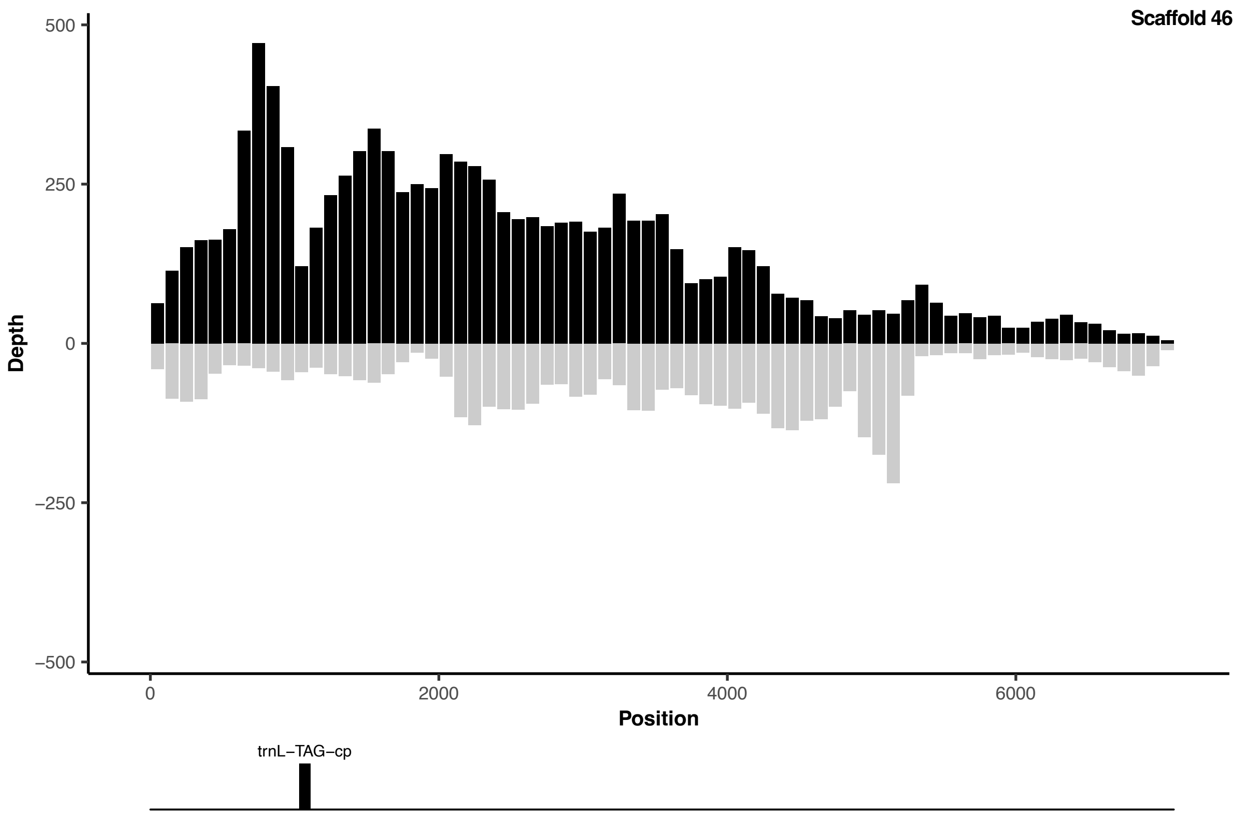
Scaffold 46
500
250
0
−250
−500
Depth
0
2000
4000
6000
Position
trnL−TAG−cp
